# Connectivity differences between Gulf War Illness (GWI) phenotypes during a test of attention

**DOI:** 10.1371/journal.pone.0226481

**Published:** 2019-12-31

**Authors:** Tomas Clarke, Jessie D. Jamieson, Patrick Malone, Rakib U. Rayhan, Stuart Washington, John W. VanMeter, James N. Baraniuk

**Affiliations:** 1 Center for Functional and Molecular Imaging, Georgetown University, Washington, DC, United States of America; 2 Department of Mathematics, University of Nebraska-Lincoln, Lincoln, Nebraska, United States of America; 3 Department of Physiology and Biophysics, Howard University College of Medicine, Washington, DC, United States of America; 4 Division of Rheumatology, Immunology and Allergy, Georgetown University, Washington, DC, United States of America; Universidad Complutense Madrid, SPAIN

## Abstract

One quarter of veterans returning from the 1990–1991 Persian Gulf War have developed Gulf War Illness (GWI) with chronic pain, fatigue, cognitive and gastrointestinal dysfunction. Exertion leads to characteristic, delayed onset exacerbations that are not relieved by sleep. We have modeled exertional exhaustion by comparing magnetic resonance images from before and after submaximal exercise. One third of the 27 GWI participants had brain stem atrophy and developed postural tachycardia after exercise (START: Stress Test Activated Reversible Tachycardia). The remainder activated basal ganglia and anterior insulae during a cognitive task (STOPP: Stress Test Originated Phantom Perception). Here, the role of attention in cognitive dysfunction was assessed by seed region correlations during a simple 0-back stimulus matching task (“see a letter, push a button”) performed before exercise. Analysis was analogous to resting state, but different from psychophysiological interactions (PPI). The patterns of correlations between nodes in task and default networks were significantly different for START (n = 9), STOPP (n = 18) and control (n = 8) subjects. Edges shared by the 3 groups may represent co-activation caused by the 0-back task. Controls had a task network of right dorsolateral and left ventrolateral prefrontal cortex, dorsal anterior cingulate cortex, posterior insulae and frontal eye fields (dorsal attention network). START had a large task module centered on the dorsal anterior cingulate cortex with direct links to basal ganglia, anterior insulae, and right dorsolateral prefrontal cortex nodes, and through dorsal attention network (intraparietal sulci and frontal eye fields) nodes to a default module. STOPP had 2 task submodules of basal ganglia–anterior insulae, and dorsolateral prefrontal executive control regions. Dorsal attention and posterior insulae nodes were embedded in the default module and were distant from the task networks. These three unique connectivity patterns during an attention task support the concept of Gulf War Disease with recognizable, objective patterns of cognitive dysfunction.

## Introduction

Between 25% and 32% of veterans from the 1990–1991 Persian Gulf War have developed chronic pain, fatigue, cognitive and gastrointestinal dysfunction, a cluster of symptoms that has been called Gulf War Illness (GWI) [1]. Diagnosis is based on the 1998 Center for Disease Control Chronic Multisymptom Illness (CMI) criteria of fatigue, mood/cognition, and bodily pain [2] and 2000 Kansas criteria with moderate or severe complaints in at least 3 of 6 categories of fatigue/sleep, neurological/mood/cognition, pain, gastrointestinal, respiratory, and skin symptom domains [3]. An important clinical finding in GWI is that physical, emotional, cognitive, or other exertion can trigger symptom exacerbations (post-exertional malaise or exertional exhaustion) [3]. This phenomenon was studied by having subjects perform 2 submaximal bicycle exercise stress tests on 2 consecutive days with functional magnetic resonance imaging (fMRI) before and after exercise [4]. Cognitive dysfunction was evaluated using the n-back working memory task [5]. This report discusses brain connectivity during the simple 0-back stimulus–response task performed before exercise as a measure of dysfunctional attention in GWI veterans compared to control subjects.

### Cholinergic hypothesis

GWI has been epidemiologically linked to Gulf War exposures to nerve agents, organophosphate pesticides, smoke from oil well fires, and the plume from munitions demolition at Khamisiyah, Iraq, in March 1991 [6–8]. Murine models of exposure to combinations of these agents mimic aspects of the human condition including the release of mitochondrial acylcarnitines and other lipids that are also elevated in the plasma of GWI patients [9]. Exposures to acetylcholinestesterase inhibitors while deployed may have caused elevated acetylcholine levels and toxic activation of muscarinic and nicotinic receptors in the central nervous system and other target cells. Cholinergic neurons innervate widespread regions of the cortex and basal ganglia and play a critical role in cognition by raising attention [10]. Much of the acetylcholine is released from axonal varicosities rather than axon terminals, and acts by diffusion to muscarinic and nicotinic receptors on surrounding neuron and glial cells. A genetic contribution to cholinergic toxicity is suggested by finding that GWI cases have a higher prevalence of butyrylcholinesterase alleles with low enzyme activity to cleave acetylcholine compared to healthy veterans [11]. However, a second study did not confirm this genetic association [12]. The role of acetylcholine in generalized brain activation and its potential toxicity provides rationale to examine attention as a component of cognitive dysfunction in GWI veterans. Another link is the cholinergic autonomic dysfunction reported in GWI [13].

### GWI subgroups based on exercise induced orthostatic tachycardia

We have reported that one third of GWI veterans developed transient postural tachycardia with no postural hypotension after exercise [4]. Loss of cholinergic vagal afferent function that decreases heart rate is one potential explanation. This post-exercise postural response was a novel physiological finding that has not been described before. Prior to exercise, GWI and healthy veterans and civilian control subjects had a normal increase in heart rate of 12 ± 2 beats per minute (ΔHR, mean ± SD) when standing up from a resting, recumbent posture. Control and two thirds of GWI participants maintained this level of ΔHR after exercise. In contrast, the other one third of GWI participants developed transient postural tachycardia only after exercise with ΔHR exceeding 30 beats per minute at several time points. This subgroup was termed the Stress Test Activated Reversible Tachycardia (START) phenotype [4]. The magnitude of ΔHR was the same as seen in Postural Orthostatic Tachycardia Syndrome (POTS). However, POTS is defined by ΔHR ≥ 30 beats per minute virtually every time a subject stands up, and with no relationship to exercise or other precipitating activity [14]. POTS may be a consequence of blunted brainstem and efferent autonomic cholinergic parasympathetic regulation to the cardiac sinoatrial node that normally maintains heart rates in the lower normal range and prevents resting tachycardia. We proposed the hypothesis that exercise caused a reduction in this efferent cholinergic reflex and allowed postural tachycardia to develop transiently in the START participants [4]. This was supported by the reduced volumes in several brainstem regions found by voxel based morphometry in the START group [4]. The regions contribute to brain arousal during tasks and stressor responses that control efferent autonomic actions [15–18].

The other two thirds of GWI participants did not have postural tachycardia or brainstem atrophy. They were termed the Stress Test Originated Phantom Perception (STOPP) phenotype based on blood oxygenation level dependent (BOLD) signals [4].

### n-back task

The cognitive task used in the MRI scanner was a challenging version of the continuous n-back verbal working memory task [4,5]. The capacity of working memory has been estimated at 4 ± 1 (mean ± SD) [19] to 7 ± 2 objects [20] in young adults, but may be different in older subjects or those with cognitive impairment [21–23]. The attention task was the simple stimulus monitoring 0-back task (“see a letter, push a button”). The 2-back task required recall of the string of letters viewed, pressing the button corresponding to the letter seen 2 previous (“2-back”), reorientation to remember the newly presented letter, update the string or chunk of letters to recall, and continue to process the remaining letters in dynamic fashion [24]. For the previous analysis, BOLD signals were contrasted between these 2 tasks. GWI subgroups had different patterns of activation in the 2-back > 0-back condition [4] and 0-back > 2-back condition [25]. For the 2>0-back condition, START activated the cerebellar vermis before exercise, but had no net activation following exercise. This supported the hypothesis that START had cognitive dysfunction with an inability to recruit brain regions for cognitive compensation. Dysfunction of the cholinergic cerebellar system could contribute to the cognitive deficits [26]. STOPP subjects had activation of the basal ganglia and anterior insulae on both days. For the 0>2-back condition following exercise, START had significant deactivation of the left dorsomedial prefrontal cortex, left precuneus, right posterior insula, right amygdala, and right thalamic nuclei. STOPP had significant clusters of deactivation in the bilateral ventromedial prefrontal cortex, bilateral precuneus, and left posterior insula. The STOPP moniker was derived from the widespread pain perceptions without demonstrable deafferentation or other causes (“phantom” experiences) in GWI and similar pattern of BOLD activation in the basal ganglia and anterior insulae as found in phantom limb and other chronic pain states [27,28]. We raised the hypothesis that the recruitment of basal ganglia and anterior insula may be a form of cognitive compensation to circumvent dysfunctional attention, reordering, and working memory cognitive processes in STOPP. Involvement of the basal ganglia was of interest because of their dense cholinergic innervation and key roles in motor, emotion, cognitive, and other brain processes relevant to GWI [29,30]. The distinctive exercise-induced changes in BOLD suggested START and STOPP had different patterns of cognitive dysfunction and utilized dissimilar patterns of brain regions for cognitive compensation.

### Connectivity

A seed region approach was used to find correlations between previously defined regions of interest (ROIs) in task and default networks. The parcellation atlas of Shirer et al. [31] provided 41 seed regions. This atlas was chosen because the ROIs and networks were defined from BOLD activation patterns during memory, music and mathematical functional tasks as well as the resting state. These a priori defined frontal, parietal and temporal regions are linked by long distance, deep white matter tracts [32]. Visual and somatosensory networks that were activated in all subjects were not included since they form a separate system of highly integrated cortical regions linked by local white matter connections beneath sulci of adjacent gyri [32]. The frontal-parietal-temporal and visual-auditory-somatomotor “rings” also differ in their patterns of gene expression [33]. This template allowed for investigation of inadequate connectivity of task regions, increased correlations between default regions that may have interfered with task performance, and regions recruited for cognitive compensation in the GWI phenotypes. For this analysis, a task system was defined by salience, basal ganglia, dorsal attention and left and right executive control networks with integrated functions [34]. The task networks set priorities for task completion, coordinate analysis of relevant visual and other sensory cues, maintain focus and content of the working memory, and track task progress. The default mode system consists of the medial prefrontal, precuneus, and parietal cortical areas. These regions have been associated with mind wandering and interospective thought, and are deactivated during externally oriented tasks. Some ROIs within the 2 systems were anticipated to have positive correlations as shown previously for the encoding and retrieval phases of working memory tasks [35].

START, STOPP and sedentary control (SC) groups have different patterns of BOLD activation during the 0-back compared to 2-back tasks when assessed as 2>0-back and 0>2-back residual conditions [4,25]. Alterations during 0-back were implied from the 0>2-back condition where the 3 groups had similar activation of anterior and posterior default mode network (DMN) nodes before exercise [25]. However, after exercise, controls had no net activation, while GWI START and “recruited” additional activation of the posterior cingulate cortex DMN. In addition, START had significant activation of bilateral middle insula. This suggested that START, STOPP and SC may have differential activation during the 0-back task.

Connectivity was determined by Pearson correlation coefficients for synchronized BOLD fluctuations between each of the selected ROIs, followed by application of standard graph theory methods [34,36–38]. The ROIs were considered to be nodes (vertices) and correlations were edges between the nodes. By its nature, connectivity studies aim to identify unique patterns of nodes and edges within groups rather than significant differences in the magnitudes of BOLD activation in individual brain regions.

“Functional connectivity” has different connotations for resting state and psychophysiological interactions in task studies [39]. In general, connectivity in resting state cannot distinguish changes related to the task from general co-activation of multiple regions by unconstrained mental activity and alterations in signal: noise ratios in nodes. The 0-back data were analysed in the same fashion as resting state and did not include additional regression to remove main effect as in psychophysiological interactions studies [39–41]. Therefore, our 0-back outcomes reflect patterns of co-activation that may be similar across all subjects and changes in functional connectivity due to the 0-back task that may be distinct in each subgroup.

## Methods

### Subjects

The protocol was approved by the Georgetown University Institutional Review Board (IRB 2009–229) and U.S. Army Medical Research and Materiel Command (USAMRC) Human Research Protection Office (HRPO #A-15547.0), and listed in clinicaltrials.gov (NCT01291758). All clinical investigations were conducted according to the principles expressed in the Declaration of Helsinki. The protocol is described to provide context, but only the data from the 0-back task before exercise are reported.

Telephone screening after verbal informed consent was performed with 209 subjects, but 105 declined to participate, 22 were ineligible by exclusion criteria, and 15 cancelled. Sixty-one participants gave written informed consent, completed questionnaires, history and physical examinations, and screening blood work. Complete exercise and MRI data was collected from 36 subjects, while 31 had partial study results (outcomes not reported here). The current study reports on 27 veterans who met Kansas [3] and CMI [2] criteria for GWI (n = 9 START, n = 18 STOPP) and 6 healthy deployed veterans from the 1990–1991 Gulf War plus 2 healthy, nonmilitary control subjects (n = 8 sedentary controls, SC). All subjects had a sedentary lifestyle with less than 40 minutes of active aerobic work or exercise per week. Additional details of the study protocol and extensive demographics, symptom profiles, and quality of life outcomes were reported elsewhere as supplementary on-line materials [4,42,43].

On Day 1, subjects had fMRI scans while performing the n-back task, followed by a submaximal exercise stress test. On Day 2, they performed the same exercise, repeated the same fMRI scanning and cognitive test, then had lumbar puncture [4].

Data are reported that were significantly different between groups for Multidimensional Fatigue [44], Center for Epidemiological Studies–Depression (CESD) [45], Beck Depression Inventory (BDI) [46], Generalized Anxiety Disorder 7 questions (GAD-7) [47], The Irritability questionnaire (TIQ) [48], Pain Catastrophizing Scale (PCS) [49], Global Interoceptive Score [50], McGill Pain Score (sensory, affective and total) [51] and quality of life by SF-36 [52]. Differences between groups were assessed by ANOVA followed by Tukey’s Honest Significant Difference with p < 0.05. Subjects were assessed for widespread pain and tenderness to thumb pressure (1990 American College of Rheumatology criteria for fibromyalgia) [53] and tenderness to pressure measured by dolorimetry (kg) [54].

### N-back experimental paradigm

The continuous n-back cognitive task tested attention and working memory [5]. Subjects practiced the task in a mock scanner until they were satisfied with their performance prior to testing in the MRI scanner [4]. Each block in the task had fixation, 0-back and 2-back components (ePrime software) (S2 Table) [55]. The block began by viewing “REST” for 0.8 sec followed by a crosshair for 9.2 sec. The “0-BACK” instruction (0.8 sec) and blank screen (1.2 sec) were followed by 9 pseudorandomized letters (A, B, C, D) seen for 0.8 sec each followed by 1.2 sec of blank screen per letter (18 sec total). “REST” was displayed for 0.8 sec followed by crosshair for 9.2 sec. The “2-BACK” instruction (0.8 sec) and blank screen (1.2 sec) were followed by 9 letters (2 sec each, 18 sec). This block was repeated 5 times. The 0-back task was performed by using both hands to press the buttons corresponding to the letters viewed (9 letters x 5 cycles = 45). For the 2-back task, subjects viewed and remembered the first 2 letters. When the 3^rd^ letter was projected, they pressed the button corresponding to the letter seen 2 (4 sec) previously. They pressed the button to recall 7 letters for a total of 35 responses in the 5 cycles. Analysis of the 2-back tasks and Day 2 were not reported here.

### MRI acquisition

Data were acquired on a Siemens 3T Tim Trio scanner equipped with a transmit-receive body coil and a commercial 12-channel head coil array as described previously [4,56]. MPRAGE parameters were: 176 sagittal slices, TR/TE/TI = 1900/2.52/900 ms, FOV = 250 mm^2^, matrix of 246x256, 1.0 mm slice thickness, and effective resolution of 1.0 mm^3^. fMRI acquisition during the n-back protocol used T2*-weighted echo planar imaging. Acquisition parameters were: 47 axial slices with a 3.2 mm thickness, TR/TE = 2500/30 ms, 90° flip angle, FOV = 205 mm^2^, 64x64 matrix for an effective resolution of 3.2 mm^3^ [4,56].

### BOLD preprocessing

Image processing and statistical analyses were performed using the Statistical Parametric Mapping 5 software package (SPM5) [57–59]. Sequential slice time correction was performed by realigning all images to the first image in order to correct for head motion artifact between scans. Spatial smoothing was performed on all scans using a Gaussian kernel of 5 mm full-width half maximum (FWHM). Realigned images were spatially normalized to fit Montreal Neurological Institute (MNI) standard stereotactic space in each subject before averaging across subjects within groups. For each subject, scans corresponding to the five 0-back BOLD time series were extracted from those corresponding to the fixation and 2-back working memory tasks and concatenated with correction for baseline wander.

ROIs from each template [31] were demarcated using MRIcron [60,61] and constructed using the Mars-BaR [62] toolkit within SPM [63]. Pearson correlations of synchronous BOLD activation between nodes (i.e., ROIs) were calculated using a custom Matlab 2014b script [64]. These correlations formed the edges between the nodes of our connectome, per previous methodology [65].

### Atlas

Double dipping and potential false positive and false negative correlations [66,67] were avoided by using the independent atlas of Shirer et al. [31] and 41 of its predefined brain seed regions (nodes) (Table 1 and S1 Table). Abbreviations incorporated the anatomical gyri and networks for anterior (SA) and posterior (SP) salience, dorsal attention network (DAN), left (LE) and right (RE) executive control and basal ganglia (BG) regions of task networks, and precuneus (PD), dorsal (DD) and ventral (VD) default mode networks (DMN) as assigned by those authors. In general, nodes in SA, SP, DAN, LE, RE and BG were considered to be the task system. Nodes in PD, DD and VD formed the default system.

**Table 1:** Abbreviations for seed regions in each a priori network defined by Shirer et al. [31]. Additional information about each ROI is shown in Supplementary Materials S1 Table.

ROI	Anatomy	Regions (Brodman Areas)
Basal Ganglia		
LBG	LBG	Left caudate and thalamus
RBG	RBG	Right caudate, putamen and thalamus
Anterior Salience Network: Anterior Insula / Dorsal Anterior Cingulate Cortex (dACC)		
SA1	LMFG	Left middle frontal gyrus (9,46)
SA2	LAI	Left anterior insula (48,47)
SA3	dACC	Anterior cingulate cortex (24,32), medial prefrontal cortex (8), supplementary motor area (6)
SA4	RMFG	Right middle frontal gyrus (46,9)
SA5	RAI	Right anterior insula (48,47)
Posterior Salience Network (Posterior Insula)		
SP1	LPI	Left supramarginal gyrus (40), inferior parietal gyrus
SP2	RPI	Right supramarginal gyrus (2,40), inferior parietal gyrus
DAN/Visuospatial Network (Frontal Eye Fields & Intraparietal Sulcus) (FEF & IPS)		
DAN1	LFEF	Left middle frontal gyrus, superior frontal gyrus, precentral gyrus (6)
DAN2	LIPS	Left inferior parietal sulcus (2,40,7)
DAN3	RFEF	Right middle frontal gyrus (6)
DAN4	RIPS	Right inferior parietal lobule (2,40,7)
Left Executive Control Network (L Dorsolateral Prefrontal Cortex / L Parietal) (DLPFC)		
LE1	LMFG	Left middle frontal gyrus, superior frontal gyrus (8,9)
LE2	LOFG	Left inferior frontal gyrus (10,45), orbitofrontal gyrus (47)
LE3	LPar	Left superior parietal gyrus (7), inferior parietal gyrus (40), precuneus, angular gyrus (39)
LE4	LITG	Left inferior temporal gyrus, middle temporal gyrus (20,37)
LE5	RCrusI	Right Crus I (cerebellum)
Right Executive Control Network (RDLPFC / R Parietal)		
RE1	RDLPFC	Right middle frontal gyrus, superior frontal gyrus (46,8,9)
RE2	RMFG	Right middle frontal gyrus (10,46)
RE3	RSMG	Right inferior parietal gyrus, supramarginal gyrus, angular gyrus (7,40,39)
RE4	RSFG	Right superior frontal gyrus (8)
RE5	LCrusI	Left Crus I, Crus II, Lobule VI (cerebellum)
Precuneus Default Mode Network		
PD1	MCC	Midcingulate cortex, posterior cingulate cortex (23)
PD2	pPrec	Precuneus, posterior (7,19)
PD3	LAG	Left angular gyrus (7,40)
PD4	RAG	Right angular gyrus (7,40)
Dorsal Default Mode Network (Posterior Cingulate Cortex / Medial Prefrontal Cortex) (PCC/MPFC)		
DD1	MPFC	Medial prefrontal cortex, anterior cingulate cortex, orbitofrontal cortex; right superior frontal gyrus (9,10,24,32,11)
DD2	LAG	Left angular gyrus (39)
DD3	PCC	Posterior cingulate cortex (PCC), precuneus (23,30)
DD4	RAG	Right angular gyrus (39)
Ventral Default Mode Network (Retrosplenial Cortex / Middle Temporal Lobe)		
VD1	LRSC	Left retrospenial cortex, posterior cingulate (29,30,23)
VD2	LMFG	Left middle frontal gyrus (8,6)
VD3	LPara	Left parahippocampal gyrus (37,20)
VD4	LMOG	Left middle occipital gyrus (19,39)
VD5	RRSC	Right retrospenial & posterior cingulate cortex (30,23)
VD6	Prec	Precuneus (5,7)
VD7	RDLPFC	Right superior frontal gyrus, middle frontal gyrus (9,8)
VD8	RPara	Right parahippocampal gyrus (37, 30)
VD9	RMOG	Right angular gyrus, middle occipital gyrus (39, 19)
VD10	RLobII	Right lobule II (cerebellum)

### Connectivity analyses

A hierarchy of criteria was used as data reduction steps to select significant edges. Pearson’s correlations between 41 nodes were calculated in the SC, START and STOPP groups, followed by conversion to Fisher’s transformed z-scores to ensure normality. Mean and standard deviations (SD) were computed for each edge in each group.

The first step was to remove edges that could not be statistically significant. Each group had its own set of unique edges: 820 edges per group x 3 group = 2460 edges. All edges with SD larger than their means were considered a null (no significant correlation) data set. The pooled null set from the 3 groups consisted of 1441 edges (0.085 ± 0.015, mean ± SD). This yielded 1019 potentially significant edges in the SC, START and STOPP groups. z-Scores (mean / SD) of each edge were compared to the null data by 2-tailed unpaired Student’s t-tests. Probability estimates (p values) were converted to False Discovery Rate (FDR p<0.01) [68] to correct for multiple comparisons between all edges and groups [69]. Data for individual groups were assessed in the same fashion and yielded the same sets of potentially significant edges and so justified using the pooled null set.

For the second step, edges in the 3 groups were constrained by requiring large effect sizes. Cohen’s d [70] was calculated to identify z-scores with a narrow range (small SD) grouped around the mean. The narrow SD eliminated edges with points that were large outliers. Large values of d improved the likelihood that significant correlations could be confirmed in future studies with reasonable samples sizes.

The threshold for Cohen’s d was selected by optimizing the number of edges that were most unique to each individual group and dissimilar from the other two groups. Jaccard indices (“intersection over union”) were calculated for each pair of groups at increasing levels of Cohen’s d to optimize dissimilarity [71,72]. For each pair of groups at each integer level of d, the number of significant edges was counted that were shared by the 2 groups (“intersection”) and divided by the total number of edges present for the 2 groups (“union”). Shannon’s entropy (log_2_) was maximized to discriminate between the 3 groups [73,74]. At each integer level of d, the fractions of significant edges were determined for each combination of the 3 groups (groups alone, pairs and all 3 groups). The fraction for each combination was multiplied by its log2 value, and the values summed. The consensus was for Cohen’s d > 1.6.

The outcome of the data reduction process was a spread sheet of 104 z-transformed correlation coefficients (edges) that were significantly different from the null set in the 3 groups, corrected for multiple comparisons, optimized for dissimilarity, and had large effect sizes that predict appropriate sample sizes for future confirmation studies. The process had the additional benefit of limiting the number of edges so that the analysis was not computationally intractable. Some edges were significant in all 3 groups, and in pairs of groups.

Correlations in each group were analyzed as graphic networks [34–37] using the python and numpy and networkx modules [75] to generate node and edge connectivity parameters. The number of connections for one node is called its degree. Nodes with high degree are more highly connected within a network. The importance of one node in a network can be assessed by drawing all possible paths (lines of edges and nodes) that connect all nodes together. Centrality is determined by the relative degree of one node compared to all others [76,77]. A node with 2 edges connected to 2 other nodes is “between” those nodes. Betweenness centrality is the ratio of the number of paths crossing through the node compared to all other nodes. High betweenness centrality indicates that information passing through the network is more likely to pass through that particular node compared to the others. The relative local importance of a node is captured by leverage centrality, the ratio of that node’s degree to the sum of the degrees of all of its adjacent nodes (range -1 to 1) [78]. A node with high leverage centrality has more links to the rest of the network compared to its neighbors. Similar indices are calculated for edges (e.g. edge betweenness centrality). These parameters were tested in general linear models to determine if they could predict group status (dependent variable).

Ball-and-spring figures depicting networks were drawn in hierarchical fashion by plotting the nodes with the highest degree and their neighbors together. The edges can be considered to be “springs” that pull connected nodes together into small “areal” maps of node communities, subnetworks or modules, and to separate them from other self-consistent components. A module is a set of nodes that has an optimal number of connections so that removing or adding a node reduces the overall connectivity of the unit [36,37]. Modularity was determined by the Louvain method for community detection [79]. Weighted modularity scores (Q) were calculated using Fisher’s transformed z-scores of Pearson correlation coefficients. Nodes were grouped to visually optimize connections within task and default systems.

Data were processed in Excel, SPSS v.22, VassarStats [80] and python [75].

## Results

### Demography and psychometrics

All of the GWI but none of the sedentary control (SC, n = 8) subjects satisfied Center for Disease Control criteria for Chronic Multisystem Illness (CMI) [2] and Kansas [3] criteria for GWI. GWI and SC groups had similar age, body mass index (BMI), and frequency of white males (Table 2). START (n = 9) and STOPP (n = 18) had significantly more impairment and greater severity ratings than SC for measures of fatigue, pain, interoception, catastrophizing, affective dysfunction, anxiety, irritability, and quality of life (significant ANOVA followed by Tukey’s Honest Significant Difference with p < 0.001) (Table 2). About half of START and STOPP subjects met 1990 American College of Rheumatology criteria for fibromyalgia [53]. START and STOPP were significantly more tender to pressure measured by dolorimetry (kg) than SC [54].

**Table 2:** Demographics and questionnaire scores for sedentary control (SC), and GWI START and STOPP phenotypes.

	SC	START	STOPP
N	8	9	18
Age (yr)	48.9 [42.8 to 55.0]	44.4 [39.2 to 49.6]	45.8 [42.3 to 49.3]
Body mass index	29.5 [25.8 to 33.2]	28.5 [24.8 to 32.2]	31.5 [27.9 to 35.1]
Male	7 (88%)	8 (89%)	13 (72%)
White	7 (88%)	7 (78%)	14 (77%)
Chronic Multisystem Illness	0 (0%)	9 (100%)	18 (100%)
Kansas criteria for GWI	0 (0%)	9 (100%)	18 (100%)
SF-36 Domains			
Physical Functioning	83.5 [69.7 to 97.3]	37.5 [19.8 to 55.2] *	45.8 [35.6 to 56.1] *
Social Functioning	71.3 [51.9 to 90.6]	15.0 [5.5 to 24.5] *	27.9 [20.1 to 35.1] *
Role Physical	67.5 [42.1 to 92.9]	0.0 [0] *	14.8 [0.02 to 29.1] *
Role Emotional	86.7 [69.3 to 104.1]	3.3 [-3.2 to 9.8] *†	37.3 [17.1 to 54.3] *
Mental Health	72.0 [64.3 to 79.7]	39.2 [25.3 to 53.1] *†	58.1 [49.8 to 64.4]
Vitality	53.5 [38.4 to 68.4]	14.5 [7.7 to 21.3] *	13.5 [7.1 to 20.4] *
Bodily Pain	65.6 [50.0 to 81.2]	17.2 [7.6 to 26.8] *	28.8 [20.2 to 37.4] *
General Health	68.2 [53.8 to 82.6]	15.7 [7.5 to 23.9] *	28.7 [18.1 to 38.7] *
Multidimensional Fatigue Inventory (MDFI)			
General Fatigue	9.5 [7.0 to 12.0]	19.3 [18.8 to 19.8] *	17.2 [15.7 to 18.7] *
Physical Fatigue	8.1 [5.7 to 10.5]	17.1 [16.0 to 18.2] *	15.4 [14.1 to 16.7] *
Reduced Activity	7.5 [5.1 to 9.9]	17.5 [14.9 to 20.1] *	16.1 [14.7 to 17.5] *
Reduced Motivation	7.6 [5.5 to 9.7]	14.9 [13.3 to 16.5] *	13.0 [11.5 to 14.5] *
Mental Fatigue	8.6 [6.3 to 10.9]	17.5 [16.1 to 18.9] *†	15.0 [13.6 to 16.6] *
CESD (depression)	8.6 [3.9 to 13.3]	38.1 [37.4 to 43.8] *†	24.8 [17.1 to 29.5] *
BDI (depression)	5.9 [2.2 to 8.6]	26.6 [20.3 to 32.9] *†	17.8 [13.5 to 22.1] *
GAD-7 (anxiety)	3.5 [0.8 to 6.2]	14.6 [12.6 to 16.6] *†	7.4 [4.9 to 10.0]
Irritability	37.8 [28.8 to 46.8]	76.0 [63.5 to 88.5] *†	54.0 [46.5 to 61.5] *
Pain Catastrophizing Score (PCS)			
Rumination	2.5 [-0.1 to 5.1]	12.9 [10.9 to 14.9] * †	6.4 [4.5 to 8.3] *
Magnification	1.3 [-0.1 to 2.7]	7.9 [6.2 to 9.6] * †	4.3 [2.7 to 5.8] *
Helplessness	3.7 [0.2 to 6.9]	15.8 [11.2 to 20.0] *†	9.8 [7.1 to 12.5] *
McGill Pain Score			
Sensory	4.9 [0.7 to 9.1]	21.3 [17.8 to 24.8] *	16.8 [14.1 to 18.5] *
Affective	0.7 [0 to 1.4]	8.7 [6.8 to 10.6] *†	5.7 [4.6 to 6.8] *
Total	5.6 [0.7 to 10.5]	30.0 [25.0 to 35.0] *	22.6 [19.4 to 25.8] *
Global Interoceptive Score (0 to 208)	20.9 [5.4 to 36.3]	103.4 [81.5 to 125.3] *†	70.0 [59.1 to 80.9] *
Fibromyalgia (1990 criteria)	13%	50%	44%
Tenderness to pressure (dolorimetry, kg)	6.5 [5.1 to 7.9]	3.2 [1.9 to 4.5] *	3.4 [2.4 to 4.2] *

Symptom severity scores indicated significantly more impairment in START and STOPP than SC by ANOVA (p < 0.05) followed by post hoc Tukey’s Honest Significant Difference

* p≤0.001 for both START and STOPP vs. SC;

^†^ p≤0.05 between START and STOPP

Mean [95% confidence intervals], (per cent of group)

This group of START had more impairment than STOPP subjects based on McGill Pain [51], Mental Fatigue [44], Center for Epidemiological Studies–Depression (CESD) [45,46], Beck Depression Inventory (BDI) [46], Catastrophizing (Rumination, Magnification, Helplessness) [49], The Irritability Questionnaire [48], and Global Interoceptive Score (Sum52) [50] (significant ANOVA followed by Tukey’s Honest Significant Difference with p < 0.05) (Table 2).

### 0-back connectivity

**Fig 1:**
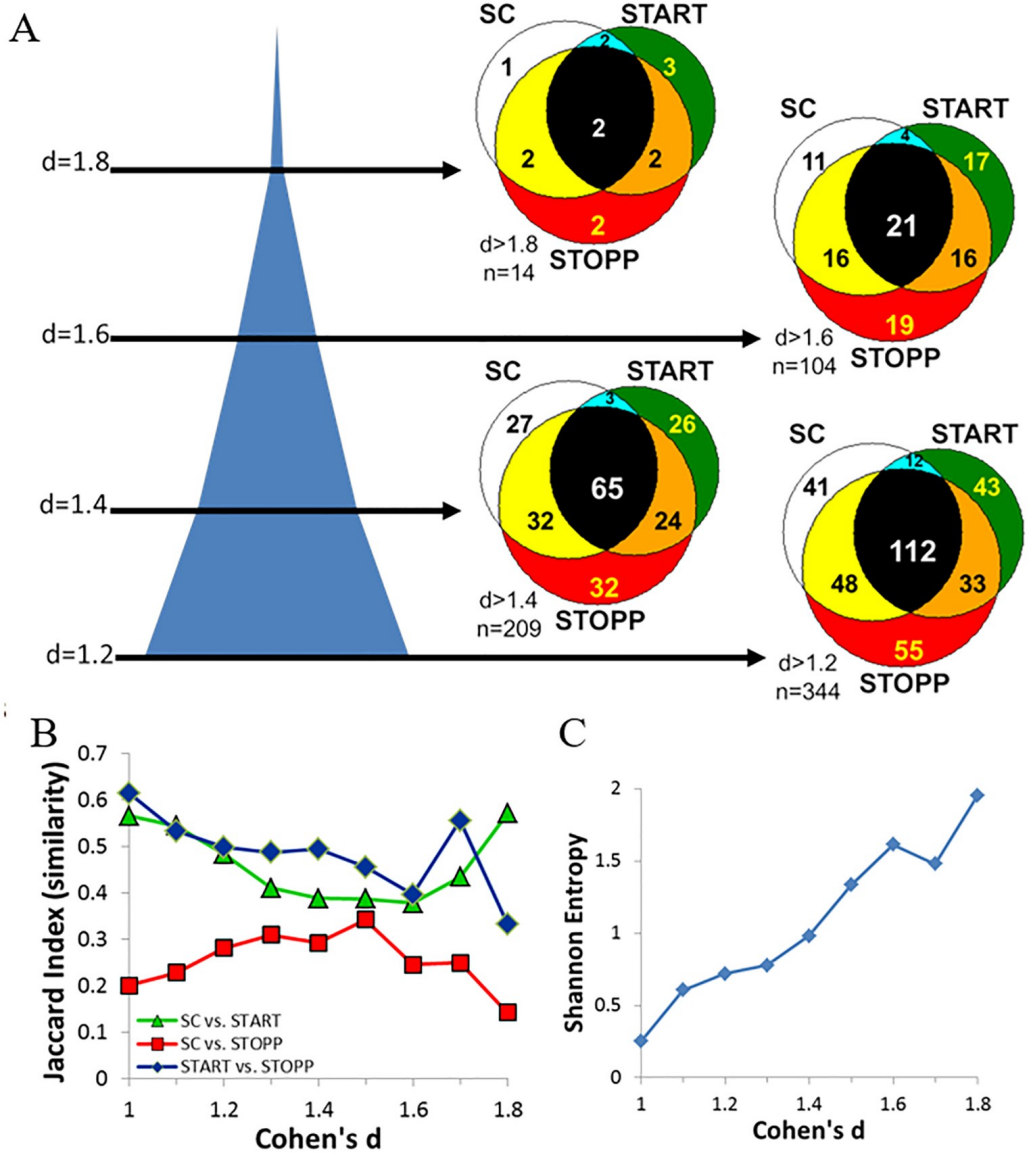
Selection of optimal Cohen’s d. (A) Cross-sections through the blue cone show the number of significant edges with FDR<0.01 at different levels of Cohen’s d. The Venn diagrams show the numbers of edges in SC (white), START (green), STOPP (red), SC&START (aqua), SC&STOPP (yellow), START&STOPP (orange) and shared by all 3 groups (black) at each level of d. (B) Jaccard indices show the similarity of each pair of groups. Similarity was lowest at Cohen’s d = 1.6. (C) The disorder, or absence of overlap between groups, was confirmed by the peak in Shannon’s entropy at the same level.

As expected, the numbers of significant edges (FDR<0.01) in SC (S3 Table), START (S4 Table), STOPP (S5 Table), and their combinations (S6 Table) were highest at Cohen’s d>1.0 and fewest at d>1.8 (Fig 1). The number of edges shared by SC&START (S7 Table) was significantly smaller than for SC&STOPP (S8 Table) and START&STOPP (S9 Table) (p < 0.0001 by log-linear 3-way contingency table [80]). This deficit illustrated the significant differences in connectivity between SC, START and STOPP. Cohen’s d>1.6 was optimal for finding high magnitude correlation coefficients with narrow standard deviations and the lowest similarity (overlap) between groups by Jaccard Indices [70,71] and Shannon’s entropy [73,74]. This large effect size implied reproducibility for future studies with significance of p<0.05, and power>80% for n≥14 per group [70].

Fisher’s transformed z-scores for the significant edges from the 3 groups were not normally distributed by Kolmogorov-Smirnov test (p<0.0001). This was not unexpected because these scores were in the upper tail of the total data set and were selected by having d>1.6. Z-scores for each group were compared by nonparametric Kruskal-Wallis test and found to be significantly different between the 3 groups (p<0.0001).

A general linear model of connectivity parameters for nodes predicted group status based on significant degree, degree centrality and leverage centrality after taking the number of ROIs into account (p<0.020). The model for edges was significant based on edge betweenness centrality (p<0.0001). Congruence coefficients (Tucker’s test [81]) indicated no similarity between SC, START and STOPP for degree, leverage centrality, and edge betweenness centrality (congruence<0.25 for each variable, with similarity inferred if 0.85 ≤ congruence ≤ 1) [82].

### Edges in individual, pairs, or shared by all groups

Significant edges were defined by Cohen’s d>1.6 and FDR<0.01, then stratified into those that were shared by all 3 groups, pairs of groups, or detected only in individual groups. These communities can be considered building blocks used to construct larger connectivity systems in the 3 individual subject groups.

SC, START and STOPP shared small communities of nodes connecting RDLPFC, DAN1 and DAN3, default system, and pairs for basal ganglia, anterior insulae and the LE1—VD2 edge (S3 Table, S1 Fig). These shared edges were depicted with highlighted backgrounds and dashed black edges, respectively, in the other figures. Edges shared by all groups may be analogous to co-activation caused by performing the 0-back task [39].

SC and STOPP shared 16 edges in 5 small communities (S4 Table, S2 Fig). Four RDLPFC and dACC nodes formed a small task community. A default community contained angular gyrus nodes clustered around the posterior precuneus (PD2).

START and STOPP shared 16 edges including a RDLPFC task community centered on dACC (SA3) and a default community of lateral parietal nodes centered on the precuneus (VD6) (S5 Table, S2 Fig).

In contrast, SC and START shared only 4 edges (S6 Table, S2 Fig).

Edges found exclusively in SC (n = 11) formed 1 community of 8 default nodes and 3 pairs of nodes (S7 Table, S3 Fig).

Edges found only in START formed 3 communities (S8 Table, S3 Fig). The large task community (12 edges) was centered on dACC (SA3, degree = 6, betweenness centrality = 0.22) and connected basal ganglia, DAN and other nodes. A chain of middle frontal gyrus nodes linked salience regions (SA4—SA1—VD2). A chain with potential behavioral relevance linked DD1 (medial prefrontal cortex) to the left inferior and middle temporal gyrus (LE4) and other nodes.

Edges found only in STOPP had 3 communities and 3 pairs of nodes (S9 Table, S3 Fig). The largest was a predominantly DMN community. A chain connected bilateral middle frontal gyrus salience nodes. The third was a task system community network of basal ganglia and rostral left (LE2) and right (RE2) frontal executive control nodes.

### SC connectivity

**Fig 2:**
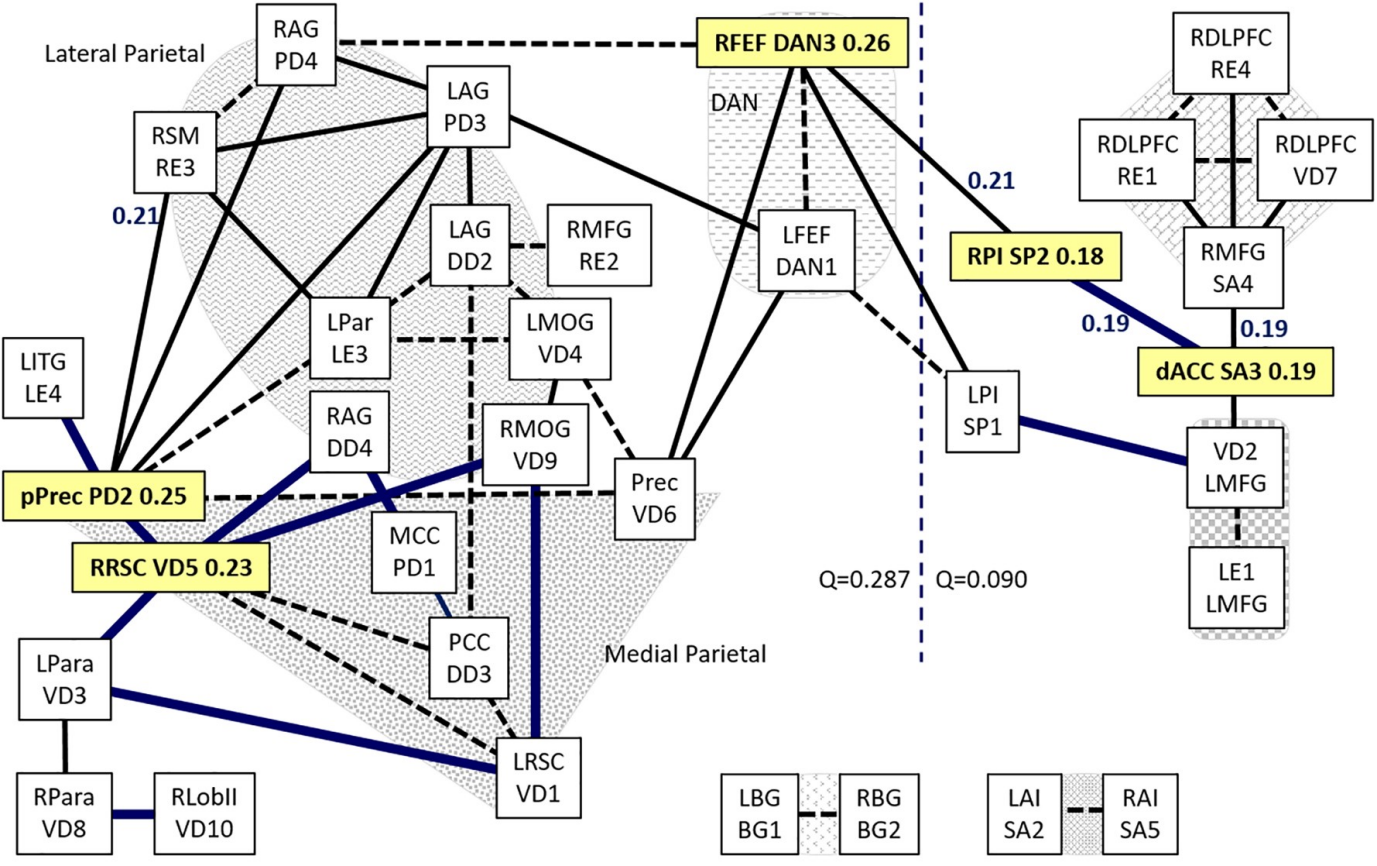
0-back connectivity in SC. All edges with d>1.6 and FDR<0.01 were plotted as a ball and spring diagram. The dotted line separated task and default modules based on the Louvain method [79] weighted by Fisher’s z-transformed Pearson correlation coefficients. Edges that were significant exclusively in SC were depicted by thick blue lines. Dashed black lines indicated edges shared by all 3 groups. Thin black lines were shared by pairs of groups. Yellow colored nodes had betweenness centrality >0.15 or were of special functional significance in SC. Edge betweenness centrality >0.15 was indicated. Grey shaded areas indicated medial parietal, lateral parietal, DAN, RDLPFC, left ventrolateral prefrontal cortex, basal ganglia and anterior insulae nodes.

The communities from SC only were combined with those shared by SC with START and/or STOPP and all 3 groups (S2 and S3 Figs). This generated a task system module linked to an extended default system module (Fig 2). Low modularity was apparent from visual inspection. Louvain modularity testing confirmed the nodes in the two modules [79].

The task system module was centered on the dACC anterior salience node (SA3, betweenness centrality = 0.19) connected to the RDLPFC community, left middle frontal gyrus, and posterior salience nodes (posterior insulae SP1, SP2). There were 2 parallel connections via posterior insulae to frontal eye field nodes in the dorsal attention network (DAN). dACC was connected to the right posterior insula (SP2) and right frontal eye field (DAN3). DAN3 was a significant bridge between task and default modules (betweenness centrality = 0.26; leverage centrality = 0.18, S10 Table). SP2 had high betweenness centrality (0.18) and high edge betweenness centrality (0.21 for SP2—DAN3 and 0.19 for SP2—SA3, S11 Table) but was more like a conduit between 2 highly connected nodes based on its low leverage centrality (-0.31). The left posterior insula (SP1) connected the left (DAN1) and right (DAN3) frontal eye fields to the left middle frontal gyrus (VD2).

The frontal eye fields were connected to the large default system module by way of the anterior precuneus (VD6), left (PD3) and right (PD4) angular gyrus nodes. Medial parietal nodes were organized around the posterior precuneus (PD2, degree = 7, betweenness centrality = 0.25) and adjacent right retrosplenial cortex (VD5, degree = 6, betweenness centrality = 0.23). These 2 adjacent nodes had the same high leverage centrality of 0.25 indicating they exerted sizable influence on information transfer compared to their neighbors in the default module. The edge between the posterior precuneus and right supramarginal gyrus had edge betweenness centrality of 0.21. SC had a unique community of ventral DMN nodes anchored by the right retrosplenium (VD5) that extended to the chain of left (VD3) and right (VD8) parahippocampus and right cerebellum (VD10), and community of left retrosplenium (VD1), left (VD4) and right (VD9) middle occipital gyri and anterior precuneus (VD6).

**Fig 3:**
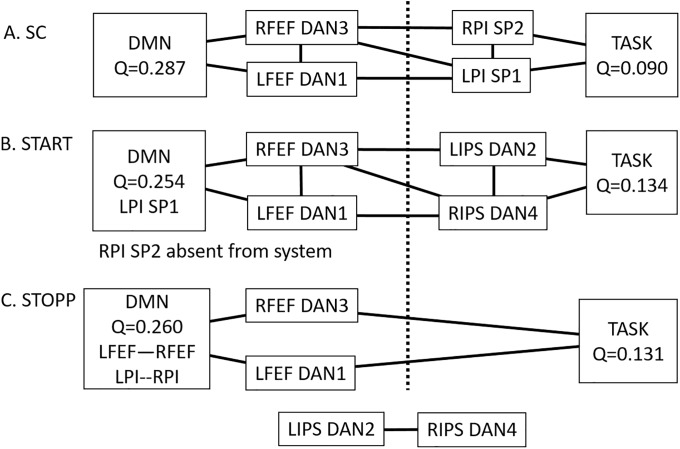
Comparison of bridging nodes and edges between groups. Modularity (Q) was calculated for default and task communities in (A) SC, (B) START, and (C) STOPP. They had 3 patterns of connecting links between DAN nodes. The arrangements of DAN and posterior salience (SP) nodes and edges were different between groups.

Louvain modularity incorporated frontal eye fields (DAN1, DAN3) in the default community (Q = 0.287) with left and right posterior insulae (SP1, SP2) in the task community (Q = 0.090) (Fig 3). The low modularity values were consistent with the small numbers of nodes, diffuse task network, and low cognitive load of the 0-back test.

Overall, SC had a task system module of left and right frontal executive, anterior and posterior salience, and DAN nodes with frontal eye fields forming the connections to the default system module (Fig 2).

### START connectivity

**Fig 4:**
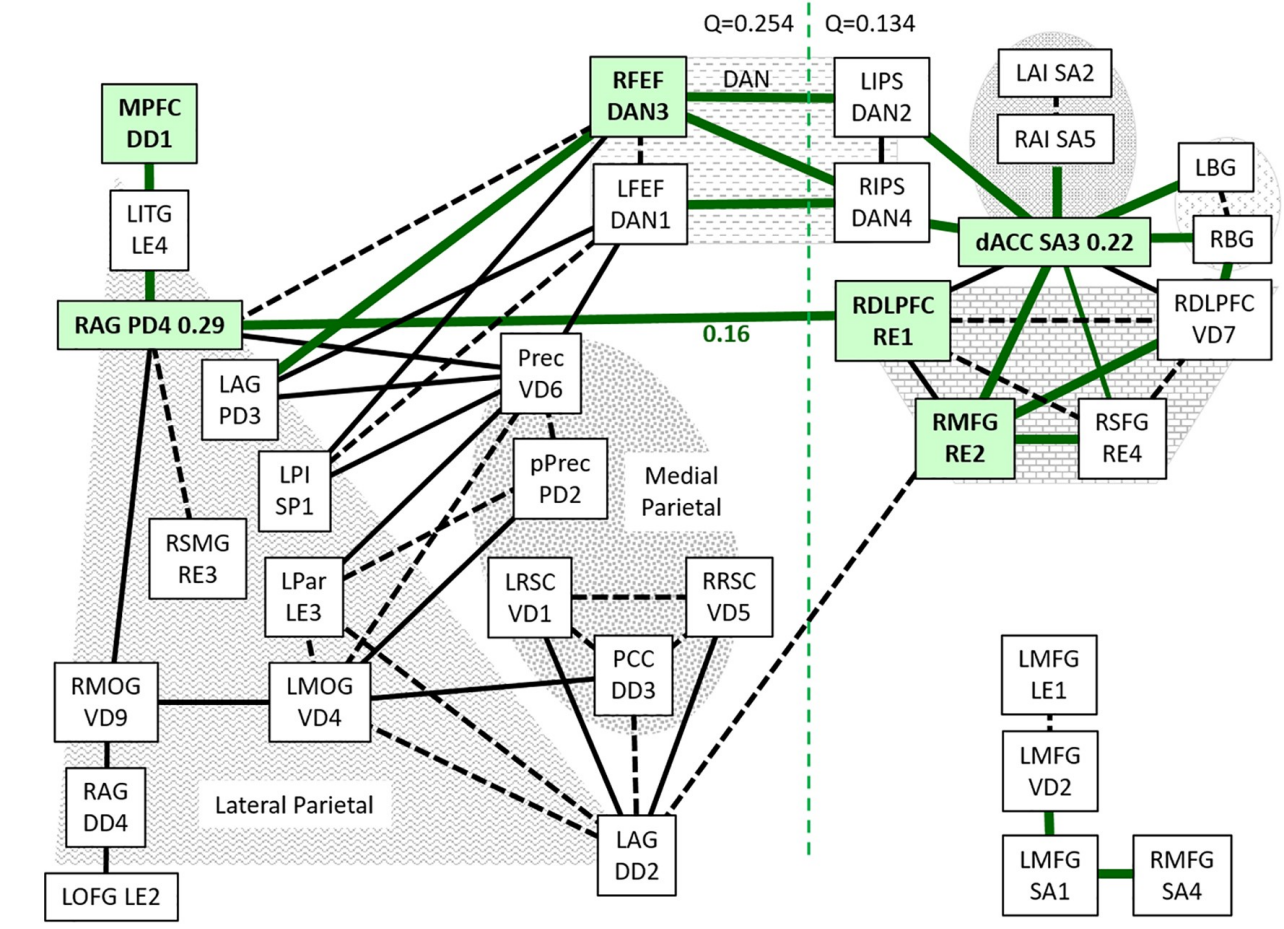
0-back connectivity in START. All edges with d>1.6 and FDR<0.01 for START were plotted as described for Fig 2. The shaded nodes had particular relevance for the START group. The RE1—PD4 edge had high betweenness centrality (0.16). The dotted line separated task and default modules.

START had a large, unique task system module centered on the dACC (SA3, degree = 9) (Fig 4). dACC had high leverage centrality (0.45) consistent with its deeply embedded position. dACC was linked to left (SA2) and right (SA5) anterior insulae, basal ganglia, RDLPFC and bilateral intraparietal sulci (DAN2, DAN4) nodes. All 4 DAN nodes were in the task module. Unlike SC, dACC was linked to frontal eye fields (DAN1 & 3) via the contralateral intraparietal sulci rather than posterior insulae. DAN3 (degree = 6) had high leverage centrality (0.23, S12 Table) as seen in SC (Table 3).

**Table 3:** Summary of qualitative differences in connectivity between SC, START and STOPP.

Feature	SC	START	STOPP
dACC (SA3)	Hub of small task system module. Degree = 3 Betweenness centrality = 0.19	Major hub of large task system module Degree = 9 Leverage centrality = 0.45	Node in system community Degree = 5
dACC to RDLPFC task community	dACC—RMFG (SA3—SA4) edge betweenness centrality = 0.19	dACC linked to 4 RDLPFC nodes	dACC linked to 5 RDLPFC nodes
dACC to LVLPFC	dACC—VD2—LE1	dACC not connected to LVLPFC community	dACC linked to LVLPFC and RDLPFC communities
dACC to basal ganglia & anterior insulae	dACC not connected to the independent LBG—RBG or LAI—RAI edges	dACC directly connected to LBG, RBG, and RAI—LAI edge	dACC indirectly connected to community LOFG (LE2), BG and AI
DAN and posterior insulae nodes	Parallel chains from dACC to posterior insulae, LFEF (DAN1) & RFEF (DAN3). Intraparietal sulci (DAN2, DAN4) not present in SC.	dACC connected to intraparietal sulci (DAN2, DAN4) then to RFEF (DAN3) Left posterior insula (SP1) in default module.	All DAN and posterior insulae nodes were in the default module, distant to the task network, or not connected
RMFG—LAG (RE2—DD2)	Terminal edge in default module. Not connected to task module.	1 of several edges connecting task and default modules	High edge betweenness centrality between task and default modules
RAG (DD4)	Default module	Default module.	Task module connecting to default module
LOFG—RAG (LE2—DD4)	Absent in SC	Peripheral edge in default module. Not connected to task module.	Task module connecting to default module
RDLPFC—RAG (RE1—PD4)	Absent in SC	Connects task and default modules with high edge betweenness centrality (0.16)	Absent in STOPP
RAG (PD4)	Connects default module to DAN3 of task module	High degree (6) and betweenness centrality (0.29) node in default module	Default module
Posterior precuneus (PD2)	Degree = 7 Leverage centrality = 0.25	Degree = 3 Leverage centrality = -0.29	Degree = 10 Leverage centrality = 0.22
Anterior dorsal precuneus (VD5)	Degree = 6 Betweenness centrality = 0.23 Leverage centrality = 0.25	Degree = 3	Degree = 3
Parahippocampi (VD3, VD8)	Default module	Absent in START	Only connected to each other
Medial parietal nodes	Linear connections with high centrality measures	Nodes not directly connected	Linear connections
Lateral parietal nodes	Random community	More connected to medial parietal than other lateral parietal nodes	Linear connections Lateral nodes linked to both anterior (VD6) and posterior (PD2) medial precuneus
Ventral default mode network (VDMN)	Connected in SC as described in atlas derived from young healthy subjects [31]	Nodes not connected No VDMN in START	Nodes not connected No VDMN in STOPP

The frontal eye fields were connected to default module nodes in the left (PD3) and right (PD4) angular gyrus, left posterior insulae (SP1) and dorsal precuneus (VD6). The right superior (RE1) and middle frontal (RE2) gyri were connected to each other (RE1—RE2) and dACC within the task module. RE1 was connected to the right angular gyrus (RAG, PD4) to form the RE1—PD4 edge (edge betweenness centrality = 0.16, S13 Table). RE2 was connected to the left angular gyrus (DD2).

The right angular gyrus (PD4) was an influential node with 6 edges (degree = 6), high betweenness centrality (0.29) and leverage centrality (0.26). An unique feature of RAG was the short chain that extended to the medial prefrontal cortex (DD1). The DD1 seed region covers the entire medial prefrontal cortex including the ventromedial prefrontal cortex that has altered activation and connectivity in depression and anxiety [83].

The dorsal anterior precuneus (VD6) was better connected (degree = 7) and more influential (leverage centrality = 0.25) than the adjacent posterior precuneus (PD2, degree = 3) that was under substantial influence from its neighbors (leverage centrality = -0.29) (Table 3).

RE2 was connected to the portion of the left angular gyrus that was in the dorsal default mode network (DD2). DD2 (degree = 6) was connected to lateral parietal (LE3, VD4), middle cingulate and retrosplenial (DD3, VD1, VD5) nodes.

A separate, independent chain was formed by left and right prefrontal nodes (LE1—VD2—SA1—SA4).

Louvain modularity was more complex than SC (Fig 3). The default community was linked to frontal eye fields (DAN1, DAN3) then intraparietal sulci (DAN2, DAN4) to the task community. Modularity calculations incorporated frontal eye fields into default mode (Q = 0.254) and intraparietal sulci into task (Q = 0.134). The bridges between default and task were different from SC because of the introduction of intraparietal sulci and shift of posterior insulae into default mode.

The key characteristics of the task system in START were the centrality of dACC in the task module, and inclusion of all 4 DAN nodes including intraparietal sulci (DAN2, DAN4) (Fig 4). DAN1 and DAN3 had 6 edges that connected to the default module. An additional edge was between right middle frontal gyrus (RE2) and left angular gyrus (DD2). The left (DD2) and right (PD4) angular gyrus nodes had high centrality in the default module. The chain of left ventral lateral prefrontal cortex was disconnected from the task module.

### STOPP connectivity

**Fig 5:**
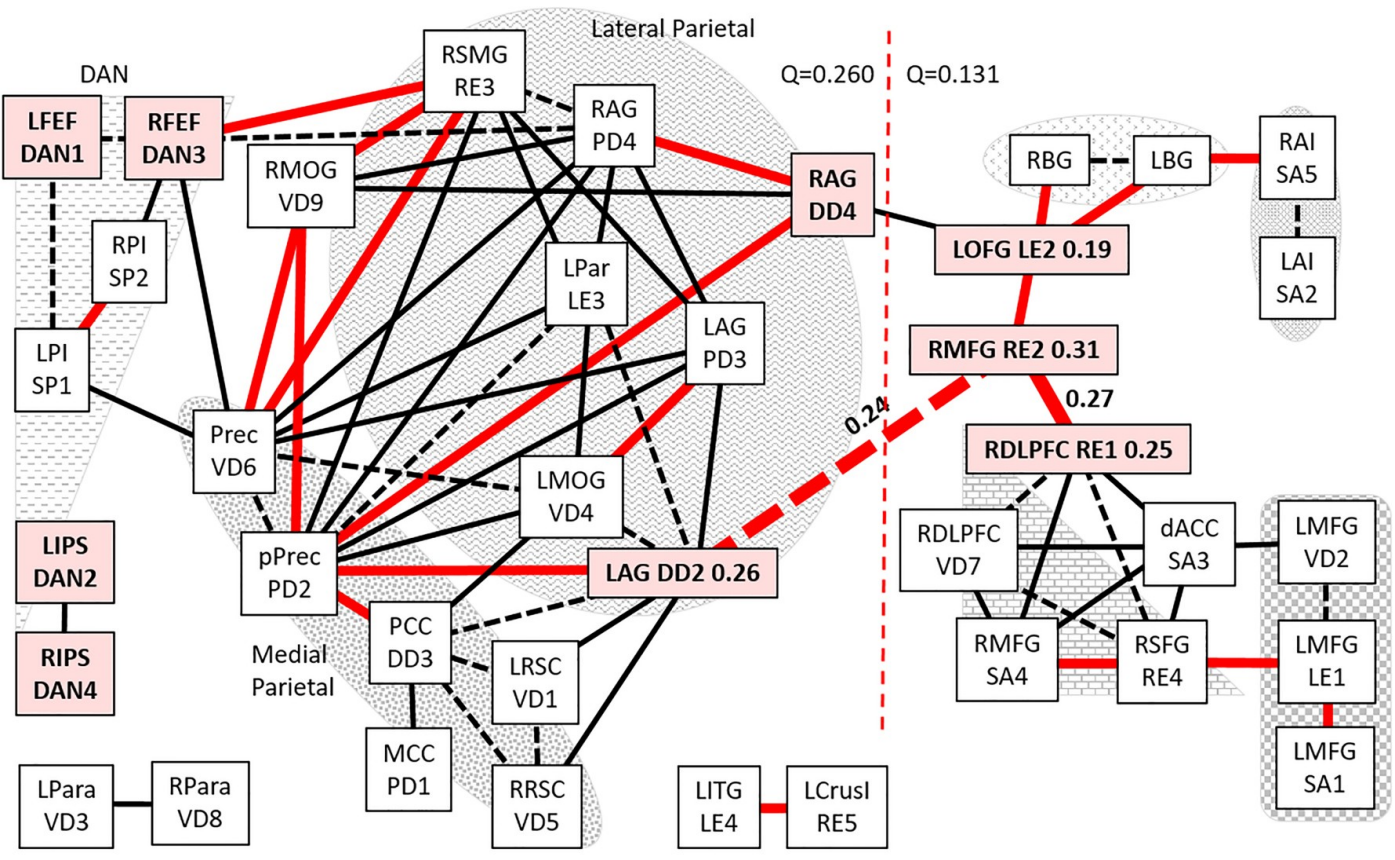
0-back connectivity in STOPP. All edges with d>1.6 and FDR<0.01 for STOPP were plotted as described for Fig 2. The shaded nodes had particular relevance for the STOPP group. High edge betweenness centrality was indicated for two edges from RE2. The dotted line separated task and default modules.

STOPP had a distinctly different task module architecture (Fig 5). Right middle frontal gyrus (RE2, betweenness centrality = 0.31, S14 Table) was a major hub connected to two separate task system subnetworks and the default module. RE2 was connected to RE1 (right superior frontal gyrus, RE1—RE2 edge betweenness centrality = 0.27, S15 Table), other RDLPFC nodes, dACC and left ventrolateral prefrontal cortex nodes. dACC was displaced from the central position it held in the SC and START task system modules. In the opposite direction, RE2 was linked to left orbitofrontal gyrus (LOFG, LE2, betweenness centrality = 0.19). LOFG was in a chain of right angular gyrus (DD4)—LOFG—basal ganglia—anterior insulae that formed an unique task subnetwork in STOPP. The LOFG (LE2)–DD4 edge was a dead end in START and absent from SC.

The right angular gyrus DD4 node formed connections to the default module by edges to its anatomically adjacent PD4 node in the right angular gyrus (degree = 8) and posterior precuneus (PD2) in the medial parietal lobe. The second bridge to the default module connected the right middle frontal gyrus (RE2) to the left angular gyrus (DD2, betweenness centrality = 0.26, RE2–DD2 edge betweenness centrality = 0.24). The RE2–DD2 edge also connected the task and default modules in START, but was a dead end in SC.

The left (DD2—PD3) and right (DD4-PD4) angular gyrus nodes and edges in the lateral parietal lobes formed an interface between the task and default systems. Each node had an edge with the highly connected medial parietal posterior precuneus (PD2, degree = 10) and/or anterior dorsal precuneus (VD6, degree = 9). DD2 (betweenness centrality = 0.26) was also connected to the posterior cingulate (DD3) and retrospenial (VD1, VD5) nodes in the medial parietal lobe. The high leverage centrality (0.22) of the posterior precuneus (PD2) was consistent with the highly intertwined DMN nodes. Four of the posterior precuneus edges were unique to STOPP.

In stark contrast to SC and START, DAN nodes in STOPP were distant from the task network (Table 3). The frontal eye fields (DAN1, DAN 3) and posterior insulae (SP1, SP2) were embedded in the default module connected to precuneus (VD6) and right supramarginal gyrus (RE3). The posterior insulae analyze pain and interoceptive stimuli; their presence within the default module may indicate distraction of attention away from task by myalgia, arthralgia, headache and irritable bowel syndrome (interoceptive perceptions) in the STOPP “phantom perception” group.

Intraparietal sulci (DAN2, DAN4) and parahippocampus (VD3, VD8) were not connected to task or default modules. This was in contrast to the connectivity of dACC to DAN2 and DAN4 in the task module of START. Connectivity of DAN to DMN nodes and their disconnection from the task modules suggested cognitive dysfunction in STOPP (Table 3).

Louvain modularity for the default community was Q = 0.260 using left and right angular gyrus (DD2, DD4) as the bridges to the task module (Q = 0.131). The bridging nodes from SC (frontal eye fields and posterior insulae) were relocated into the default community (Fig 3). The intraparietal sulci utilized in START were not incorporated in the STOPP task or default communities.

STOPP was characterized by the large bifurcated task module of RDLPFC and LVLPFC nodes connected through RMFG (RE2) to the left orbitofrontal gyrus (LOFG, LE2) chain with basal ganglia and anterior insulae. The RE2—LE2 edge was connected to left and right angular gyrus nodes, then to the medial parietal lobe of the default module. DAN and posterior insulae nodes were distant from the task module in STOPP (Fig 5).

### Dorsal precuneus

DMN nodes in the dorsal precuneus can be recruited into task systems during n-back testing in contrast to the ventral precuneus [84]. We proposed that the number of connections (degree) for superior (VD6) and posterior (PD2), but not the inferior (DD3), precuneus seed regions would be an indicator of task difficulty or recruitment of cognitive reserve regions in GWI. Degree for VD6 increased from 3 in SC to 7 in START and 9 in STOPP. Degree for PD2 was 6 in SC, 3 in START, but increased to 11 in STOPP. As anticipated, the ventral DD3 region had similar degrees in SC (4), START (4) and STOPP (6). The left (DD2, PD3) and right (DD4, PD4) angular gyri were similarly investigated and again STOPP had more total edges (26) than SC (16) and START (17). The angular gyri are important for conceptualization and control of attention shifts in time and space [85–87]. The increased connectivity of superior and dorsal precuneus regions during the 0-back task suggested dysfunctional attention in STOPP.

### Middle frontal gyrus

Frontal pole regions (BA10) have been suggested to integrate and coordinate multiple discrete cognitive operations during complex n-back tasks in control subjects [5]. Recruitment of ventrolateral prefrontal cortical regions such as the caudal middle frontal gyri has been associated with increasing cognitive load and greater task difficulty [88]. Therefore, we ranked the connectivity of the rostral right middle frontal gyrus (RE2) using degree (number of edges) and found START (5) > STOPP (3) > SC (1). The number of edges between seed regions in the middle frontal gyri (SA1, SA4, LE1, LE2, RE1, RE2, VD2) were ranked STOPP (14) > START (8) > SC (3). The increase in connectivity between middle frontal gyrus areas was taken as an indicator of ROI recruitment for cognitive compensation during the 0-back task in these phenotypes of GWI neuropathology.

### Correlation between groups

The value of connectivity is that relationships between regions of activation are identified by patterns of correlations rather than significant differences in their magnitudes between groups. Only the edge between LPar—LAG (LE3-PD3) had significant differences between groups (lower in START than control) and z-scores greater than 1 (S16 Table). Data from all significant edges are found in S17 Table.

## Discussion

The most important finding was the distinctly different connectivity patterns during the 0-back attention task before exercise for SC (Fig 2), START (Fig 4), and STOPP (Fig 5). This establishes a baseline difference between GWI and control subjects, and some as yet unclear principle related to START—STOPP cardiovascular status. These preliminary data are discussed in the context of other disorders in the differential diagnosis of GWI in order to generate new hypotheses for GWI pathology, and plans for future studies.

### Connectivity shared by SC, START and STOPP

SC, START, and STOPP had unique overall patterns of connectivity during the 0-back task. These were built from community features shared by all 3 groups (S1 Fig), pairs of groups (S2 Fig), or that were unique to individual groups (S3 Fig). Edges shared by all 3 groups may represent co-activation of brain regions rather than specific 0-back task-related functional connectivity [39].

A task system community incorporated the selective attention and salience system of anterior insula (SA2, SA5) with executive control RDLPFC [89]. RDLPFC has been reported to have 2 distinct subregions [90]. The posterior-dorsal subregion may conform to the junction of the right inferior frontal sulcus and central sulcus that has been associated with the detection of salient stimuli, action execution and working memory, and has increased connectivity with bilateral intraparietal sulci of the dorsal attention network (DAN2, DAN4) [91]. The anterior-ventral subregion of the right inferior frontal gyrus was activated during successful active inhibition of motor responses and had increased connectivity to the anterior cingulate cortex (dACC) and primary motor areas [92,93].

Regions of the left lateral prefrontal cortex have specific roles in working memory tests [5,88,94,95]. Protocol designs lead to “task set” effects with activation of left rostral lateral prefrontal cortex and left superior parietal lobule–intraparietal sulcus. Verbal tasks activated Broca’s area (left BA44/45, approximately LE2 [31]) while nonverbal tasks had greater activation of caudal middle and superior frontal gyri. Memory for stimulus identity was localized to ventral premotor areas in the caudal inferior frontal gyrus. Memory for stimulus location was associated with caudal superior frontal gyrus (dorsal premotor cortex). More difficult tasks with higher cognitive demands added activation of the right caudal inferior prefrontal gyri [96], pre-supplementary motor area, inferior parietal cortex and superior parietal lobule. Activation of the rostral ventrolateral and dorsal anterior insula [97] may indicate error monitoring [98]. These patterns were derived from healthy young adults [31] while the current study had older adults with complaints of cognitive dysfunction.

Activation of the right anterior insula (SA5) [5,88,95] was reminiscent of the right dominant selective attention system [89] and ventral attention network (VAN) [99] that has a second hub centered around the right temporoparietal junction (TPJ) [89], caudal supramarginal gyrus (BA40) and posterior superior temporal gyrus (BA22) [100]. RDLPFC nodes (RE1, RE4, VD7) were connected in all groups during the 0-back task. However, only START and STOPP integrated the anterior insulae into their task system modules suggesting that attention and active monitoring were recruited for cognitive compensation during the 0-back task.

Left ventral and lateral prefrontal cortex communities were part of the task system modules in control and STOPP, but formed an independent, detached chain in START.

Dorsal attention network regions of the frontal eye fields (DAN1, DAN3, BA6) and salience network posterior insulae (SP1, SP2) were connected in all 3 groups and may provide visual and somatic orientation. The region of the right posterior insula and supramarginal gyrus (SP2) may articulate “mouth” behaviors such as oral “mouthing” of letters to aid in working memory [94]. Adjacent regions of the left and right supramarginal gyri and BA40 (SP1, SP2, LE3, RE3) may participate in short term storage of information (LE3) and rapid switching of attention [5]. Visuospatial orientation may have been reinforced by middle occipital (VD4, VD9), middle cingulate (PD1) and retrosplenial (VD1, VD5) activation [101].

DMN nodes shared by the 3 groups included medial parietal (PD2, VD1, VD5, DD3, VD6) and left angular gyrus (DD2) nodes that may have (i) task-relevant episodic memory functions during the 0-back task [102], (ii) an “active” but non-task contemplative state of self-generated thought for decision making, internal representations of reconstructed or imagined situations, or (iii) an off-focus, internally directed mind-wandering state that interfered with completion of the 0-back task [103]. The locus coeruleus norepinephrine system may modulate these states of mind wandering by adaptively controlling the transition between exploring new avenues and exploiting existing ones (“exploration–exploitation tradeoff”) [104]. This implies that dysfunction of adrenergic innervation may alter attention in GWI, and that appropriate adrenergic therapies may correct the deficits.

### Connectivity for sedentary controls (SC)

SC had a compact task system module incorporating RDLPFC, dACC, left ventrolateral prefrontal cortex (LE1, VD2) and frontal eye fields (DAN1, DAN3). The SC default system incorporated middle and posterior cingulate gyri, precuneus and angular gyri in a densely integrated module. Only SC incorporated the chain of parahippocampus to right cerebellum (VD3—VD8—VD10) into the default module. Left parahippocampal connectivity during the resting state has been noted in controls but not Chronic Fatigue Syndrome [105] suggesting the absence of this link may be an indicator of cognitive dysfunction in nociceptive, interoceptive and fatiguing illnesses. An open question is whether control subjects utilized long term memory, memory of “place” in a list using hippocampal place cells or other recall mechanisms, processing and error checking by comparison to memories of letters as font or typeface, or letters visualized as a “chunk” of information [24,106].

### Connectivity in STOPP

In contrast to the integration of SC networks, STOPP had a bifurcated task system of salience and RDLPFC communities linked via RMFG (RE2). The large default system module was centered on cingulate, precuneus and lateral parietal nodes (Fig 5). An unique finding in STOPP was that DAN nodes were not linked to the task module, but were embedded in the DMN module. This suggested that dysfunction of attention systems involved in maintaining focus and concentration during simple cognitive tasks may contribute to cognitive disability in STOPP subjects. The left orbitofrontal gyrus (LE2) linked the default module to the salience—executive control task system module, and implicated effort and reward as an important arbiter of cognitive processes in STOPP subjects [107]. Connectivity of LE2 to the left and right basal ganglia suggested active decision making during the simple button pressing 0-back task in STOPP [108]. A more nuanced analysis of sequential activation of the ventral striatum, putamen, head of the caudate nucleus, and body of the caudate during the 0-back stimulus (“see a letter”), preparation of response, motor response (“press a button”), and feedback phases will require a more detailed model of basal ganglia seed regions [109].

Overall, STOPP was characterized by increased connectivity in the left and right middle frontal gyri, dorsal precuneus, angular gyri and temporoparietal junctions compared to SC and START. These changes were interpreted as cognitive compensation in the STOPP group (Table 3). The fracturing of DAN connections away from the task module may indicate disruption of attentiveness contributing to cognitive dysfunction in STOPP.

### Connectivity in START

START was distinctive with its large task system module of salience, basal ganglia, DAN and RDLPFC nodes and edges that were presumably recruited as cognitive compensation (Fig 4). START had more DAN (n = 12) and dACC (SA3, n = 9) edges than SC (n = 7 and n = 3, respectively) and STOPP (n = 7 and n = 5, respectively), indicating that START and STOPP had different pathways for attention and cognitive compensation.

The unique edge of ventromedial prefrontal cortex (DD1) to left lateral temporal lobe (LE4) in START will need to be investigated further given the chronic pain, systemic hyperalgesia, and subjective complaints of depression, mental fatigue and catastrophizing in this cohort (Table 2). Previous studies show the extensive anterior DMN seed region has pregenual (pACC) and subgenual anterior cingulate cortex (sACC) subdivisions with distinct functions and cytoarchitectonic, receptorarchitectonic, cortical and subcortical connectivities [110]. The pregenual medial prefrontal cortex has a rostral rim that evaluates psychological stressors and is linked to basolateral amygdala, and dopamine-rich nucleus accumbens and ventral tegmental area [16]. An inner pregenual rim is more responsive to physical threats and is linked to the central nucleus of the amygdala, solitary tract, locus coeruleus and sympathetic nervous system effector regions [16]. Subgenual area s24 of sACC was associated with sadness [83] which is relevant to the significantly higher scores on depression questionnaires for START than SC and STOPP. Resting state activation studies in major depression have found reduced connectivity of the sACC in subjects with the highest symptom severity scores [111,112]. Alternatively, connectivity of DD1 may be explained by activation of sACC area s32 that was associated with fear processing and executive control network activation during tasks that did not have an emotional component. Depression subjects have increased connectivity between the ventromedial prefrontal cortex (DD1) and paraventricular nucleus of the thalamus [83]. However, thalamic and amygdala seed regions were not used here. The inability to discover novel connectivity was a limitation of studying correlations between a priori defined seed regions. Another difference between major depression and START was the high connectivity between the dACC (SA3) and right anterior insula (SA5) in START in contrast to low connectivity for the dACC to left (SA2) and right (SA5) anterior insulae in depression [112,113].

Anxiety proneness and lower perceived control were associated with heightened activity in dorsal anterior insula in contrast to diminished insula activity in depressed individuals [114]. This was consistent with the elevated anterior insula connectivity, Generalized Anxiety Disorder (GAD-7), rumination, magnification and helplessness scores that were significantly higher in START than SC and STOPP (Table 2). These relationships may point towards principal distress disorder (generalized anxiety disorder, GAD) rather than principal fear disorders such as specific phobia, social phobia or separation anxiety disorder in GWI [115]. If so, connectivity of the dACC and right anterior insula to amygdala, midbrain and sympathetic nervous system may contribute to arousal and autonomic anxiety-like symptoms as part of “freeze–fight–flight–faint” sympathetic responses to perceived threats [16–18,116–118].

In addition, the ventral anterior insula is structurally connected to the basolateral amygdala; the connectivity explained 40% of state anxiety variance across subjects and was correlated with increased axial diffusivity of the connecting white matter tract [119]. This connection modulates sympathetic activity related to emotional states [120,121] suggesting that dysfunctional threat appraisal, heightened irritability, anxiety, startle responses and sense of loss of control in START (Table 2) may be due to dysregulated dACC, right anterior insula, amygdala and sympathetic efferent activity, and white matter dysfunction with increased axial diffusivity [51,114,122,123]. The close association of dACC and anterior insulae in START (SA3–SA5–SA2, Fig 3) may also be related to central perception of interoceptive, somatosensory and other inputs that act as distractors, interrupt focus on task, and impair cognitive function [124]. Painful stimuli activate the ventral portion of sACC area 33 and posterior insulae, components of the sensorimotor network [110,125] that were activated in fibromyalgia [126]. This was relevant to the chronic pain and systemic hyperalgesia in START (Table 2). The posterior insula (SP1) was connected to the frontal eye fields suggesting a role in surveillance for visual cues and attention.

dACC and anterior insula were connected in START, and are structurally connected by thick diameter, rapidly conducting von Economo neurons [127]. There are 50% more von Economo neurons in the right than the left hemisphere which may lead to augmented sympathetic activation. Selective destruction of von Economo neurons in early stages of frontotemporal dementia implies that they are involved in empathy, social awareness, and self-control [128]. By analogy, von Economo neuron dysfunction may contribute to anxiety and irritability (Table 2) in START neuropathology.

The relatively recent evolution of von Economo neurons [127] and the right temporal-parietal junction of the ventral attention network [100] indicates humans have more sophisticated brains than rodents and other model species. This must be taken into account when extrapolating from mice to men. For example, low dose nerve agents, other acetylcholinesterase inhibitors, mild traumatic brain injury and epilepsy induce anxiety states that may be the results of long term dysfunction of γ-aminobutyric acid (GABA), cholinergic muscarinic and nicotinic receptor, and other neurotransmitter systems in the basolateral amygdala and connected brain regions [129–132]. Comparable molecular changes may have been induced by days to weeks of exposures to low dose, nonlethal, irreversible nerve agents and reversible pyridostigmine bromide in genetically susceptible veterans in the Persian Gulf. These acetylcholinesterase inhibitors may have caused subacute to chronic acetylcholine neurotoxicity that led to GWI [7–9]. The increased prevalence of butyrylcholinesterase alleles with low acetylcholinesterase enzyme activities in GWI relative to nondeployed subjects supports investigation of this cholinergic neurotoxicity hypothesis [6].

### Limitations

Correlation of activation in prespecified seed regions [31] has technical limitations [40]. Large nodes such as the ventromedial prefrontal cortex (DD1) contains subregions that mediate a wide diversity of functions [31,83,110]. Connectivity between anatomically adjacent nodes, such as those in the angular gyrus [85] and temporoparietal junction, may indicate activation of a single larger region that happened to be subdivided in this atlas. More granular parcellation [133] will improve the matching of anatomical loci to cognitive, emotional and other functions, but the increased numbers of regions and interactions will increase the computation demands of the analysis. The atlas used here [31] was based on task and resting state BOLD, and may be inconsistent with systems derived from resting state and other n-back and working memory studies in young and elderly controls, Gulf War Illness, CFS, fibromyalgia and related conditions [4,5,88,94,105,134]. Other atlases outline ROIs that overlap with the Shirer atlas, and may group some regions into alternative networks by independent component analysis or other methods [135,136]. The unique connectivity patterns in START, STOPP and SC suggest that disease- and task-specific parcellation schemes may be needed in future studies of cognitive disabilities and compensation in START and STOPP. Masks of activated regions defined by independent component analysis from START and STOPP subjects may help refine the regions with 0-back dysfunction and may be used as a “training set” in verification studies using new “test sets” of subjects. This would avoid the risk of “double dipping” [66,67]. Undirected whole brain connectivity studies are another alternative.

Correlations were stated for only 41 of the 90 regions in the atlas of Shirer et al. [31]. In preliminary analysis, data from somatomotor, visual, language and auditory systems had small magnitude Fisher z-scores for Pearson correlation coefficients that were not statistically different between groups and were excluded by the data reduction strategy because of small Cohen’s d values (d < 0.6). A number of small ROIs in DAN (occipital cortex, cerebellum), posterior salience (thalamus, midcingulate, precuneus), and dorsal DMN (hippocampus, thalamus, midcingulate cortex) were not included in this analysis. If these ROIs had been included in the analysis, it is possible that they would have had significant correlations with other members of their respective networks that may have provided additional information about cognitive dysfunction in GWI subgroups. The absence of these potential additional correlations does not diminish the significant relationships already identified in control, START and STOPP groups. Instead, they emphasize the need to use these initial findings to define the dysfunctional network architectures during the 0-back and 2-back tasks by using larger samples of GWI veterans and controls. The nodes and edges identified in this initial study provide a guide to estimate sample sizes for future investigations, define ROIs for seed region analysis, and infer n-back related networks that may be specific for START and STOPP subgroups of GWI veterans.

The number of total correlations was large, but there was significant data reduction by using FDR<0.01 and optimizing the Cohen’s d threshold for data inclusion by maximizing the dissimilarity between group data using Jaccard indices [70,71] and Shannon entropy [74,75]. These 3 indices provided a rationale for data reduction as an alternative to selecting some arbitrary cut-off value. This strategy excluded edges with large standard deviations that would indicate wide ranges of z-scores due to outliers, highly skewed or bimodal data distributions. Using lower values of Cohen’s d would have increased the number of edges that were shared between groups and so reduced the ability to classify functionally significant differences between groups. Higher levels of Cohen’s d would have identified fewer nodes and edges and limited the likelihood of finding scientifically interesting differences. Few edges had significantly different correlation coefficients between groups, but it was our aim to identify associations through connectivity (Table 3) [137] and correlation [138] rather than illness-specific differences for particular nodes and edges (S16 Table). Connectivity analysis is fundamentally different because it infers serial and parallel pathways of brain information flow rather than regional differences in hemodynamic BOLD activation [40].

Significant differences were found between SC, START and STOPP based on differences in nodes and edges within the task and default system modules, patterns of connectivity, and connectivity parameters. Other methods such as Network Based Statistics [138] follow a comparable pipeline of node selection, pairwise association of all nodes, data transformation, data reduction by thresholding to define sparse graphs, and correction for multiple comparisons and family wise error using False Discovery Rate (FDR) [68], but instead use permutation methods to identify significant differences in nodes, edges and network components between groups. This method has the greatest benefit when p-values are marginal due to low signal-to-noise ratio, but has the disadvantage that it can only test whether an entire network component is different between groups, and cannot assess significance for individual edges. Permutation methods may lead to overfitting of the data to models and false positive inferences.

General linear models and psychophysiological interactions [39–41] provide another alternative method of analysis that may have been able to refine the results for the 0-back state by regressing out main effects. This and other approaches [41,138,139] will be considered for future incorporation into a priori statistical plans to evaluate larger samples and data sets for structured comparisons of resting state, fixation / instruction, 0-back and 2-back time series data.

Louvain modularity scores were weighted by z-scores rather than node degree. Q scores range from -1 to +1, but were relatively low for these modules (range 0.287 to 0.090 found in SC). This was consistent with the small network sizes and low cognitive load. Higher modularity was predicted for the higher cognitive load 2-back task, and for analysis of larger networks such as those employing smaller values of Cohen’s d.

The small numbers of subjects tested in this cross-sectional analysis require that larger studies be performed to confirm these findings. To that end, Cohen’s d effect sizes were used as a criterion to select correlations with a high probability of being reproducible. The 0-back task is a stimulus matching test that requires attention to the screen, and may not predict effects at higher cognitive loads or in the resting state. However, these limitations do not lessen the importance of finding significantly different connectivity patterns between control subjects and the START and STOPP phenotypes of GWI (Table 3).

### Conclusion

START and STOPP phenotypes of GWI have significant deviations in the patterns of connectivity and possible co-activation during the 0-back attention task compared to each other and sedentary control subjects. This provides objective evidence of cognitive disability in GWI veterans.

## Supporting information

S1 Table Reference brain regions of interest (ROIs) and networks. The anatomical regions that were activated synchronously as networks were shown with their original network classification [31] and the abbreviations used here (ROI). Corresponding Brodman Areas ({BA}) and approximately aligned BrainMap Intrinsic Connectivity Networks (ICN) [94] are given for further reference. ROIs were numbered for each network (reproduced with permission from Oxford University Press). Networks were listed as basal ganglia (BG), anterior (SA) and posterior salience (SP), Dorsal Attention Network (DAN), left and right executive control (LE, RE), and precuneus (PD), dorsal (DD), and ventral (VD) default mode networks. ROIs in the Shirer networks were large and irregularly shaped making it difficult to estimate Montreal Neurological Institute coordinates [140] that would capture the center of mass for each region. These coordinates were displayed as ball and spring plots [141] in S1–S3 Figs to depict approximate network connections.
(DOCX)

S2 Table Timing for 0-back and 2-back paradigm in ePrime software [55]. (DOCX)

S3 Table Nodes and edges shared by the SC, START and STOPP groups. All significant edges in individual groups, pairs of groups, and the entire group were tabulated with the average Fisher’s z-transformed Pearson’s correlation coefficients, standard deviations, Cohen’s d (d > 1.6), and Student’s t-test (FDR < 0.01). Edges were arranged by connected modules (S1 Fig). The anatomical location, estimated approximate Montreal Neurological Institute (MNI) coordinates from the original reference [31], and most closely aligned BrainMap Intrinsic Connectivity Network (ICN) [94] were shown for each node.
(DOCX)

S4 Table Nodes and edges shared by the SC and STOPP groups. All significant edges in individual groups, pairs of groups, and the entire group were tabulated with the average Fisher’s z-transformed Pearson’s correlation coefficients, standard deviations, Cohen’s d (d > 1.6), and Student’s t-test (FDR < 0.01). Edges were arranged by connected modules (S2 Fig). The anatomical location, estimated approximate Montreal Neurological Institute (MNI) coordinates from the original reference [31], and most closely aligned BrainMap Intrinsic Connectivity Network (ICN) [94] were shown for each node.
(DOCX)

S5 Table Nodes and edges shared by the START and STOPP groups. All significant edges in individual groups, pairs of groups, and the entire group were tabulated with the average Fisher’s z-transformed Pearson’s correlation coefficients, standard deviations, Cohen’s d (d > 1.6), and Student’s t-test (FDR < 0.01). Edges were arranged by connected modules (S2 Fig). The anatomical location, estimated approximate Montreal Neurological Institute (MNI) coordinates from the original reference [31], and most closely aligned BrainMap Intrinsic Connectivity Network (ICN) [94] were shown for each node.
(DOCX)

S6 Table Nodes and edges shared by the SC and START groups. All significant edges in individual groups, pairs of groups, and the entire group were tabulated with the average Fisher’s z-transformed Pearson’s correlation coefficients, standard deviations, Cohen’s d (d > 1.6), and Student’s t-test (FDR < 0.01). Edges were arranged by connected modules (S2 Fig). The anatomical location, estimated approximate Montreal Neurological Institute (MNI) coordinates from the original reference [31], and most closely aligned BrainMap Intrinsic Connectivity Network (ICN) [94] were shown for each node.
(DOCX)

S7 Table Nodes and edges in SC group. All significant edges in individual groups, pairs of groups, and the entire group were tabulated with the average Fisher’s z-transformed Pearson’s correlation coefficients, standard deviations, Cohen’s d (d > 1.6), and Student’s t-test (FDR < 0.01). Edges were arranged by system modules (S3 Fig). The anatomical location from Shirer et al. [31], approximated center of mass in Montreal Neurological Institute (MNI) coordinates [140], and most closely aligned BrainMap Intrinsic Connectivity Network (ICN) [94] were estimated for each node.
(DOCX)

S8 Table Nodes and edges in START group. All significant edges in individual groups, pairs of groups, and the entire group were tabulated with the average Fisher’s z-transformed Pearson’s correlation coefficients, standard deviations, Cohen’s d (d > 1.6), and Student’s t-test (FDR < 0.01). Edges were arranged by connected modules (S3 Fig). The anatomical location from Shirer et al. [31], approximated center of mass in Montreal Neurological Institute (MNI) coordinates [140], and most closely aligned BrainMap Intrinsic Connectivity Network (ICN) [94] were estimated for each node.
(DOCX)

S9 Table Nodes and edges in STOPP group. All significant edges in individual groups, pairs of groups, and the entire group were tabulated with the average Fisher’s z-transformed Pearson’s correlation coefficients, standard deviations, Cohen’s d (d > 1.6), and Student’s t-test (FDR < 0.01). Edges were arranged by connected modules (S3 Fig). The anatomical location from Shirer et al. [31], approximated center of mass in Montreal Neurological Institute (MNI) coordinates [140], and most closely aligned BrainMap Intrinsic Connectivity Network (ICN) [94] were estimated for each node.
(DOCX)

S10 Table Connectivity parameters for nodes for sedentary control group (SC). (DOCX)

S11 Table Connectivity parameters for edges in the sedentary control (SC) group. (DOCX)

S12 Table Connectivity parameters for nodes in START phenotype. (DOCX)

S13 Table Connectivity parameters for edges in START phenotype. (DOCX)

S14 Table Connectivity parameters for nodes in STOPP phenotype. (DOCX)

S15 Table Connectivity parameters for edges in STOPP phenotype. (DOCX)

S16 Table Edges with significantly different Fisher z-transformed Pearson correlation coefficients between groups (mean ± SD). (DOCX)

S17 Table Raw data. **Fisher z-transformed Pearson correlation values for all subjects and edges**. Excel file attached on-line.
(7Z) Click here for additional data file.

S1 Fig Communities of nodes and edges shared by SC, START and STOPP. Connectivity maps show small communities (“cores”) for (a) RDLPFC task, (b) DAN1 and DAN3, (c) default system, and (d) 3 additional pairs of nodes. These shared edges were indicated by dashed lines on the other figures and were detailed in S6 Table. (e) Ball and spring models of nodes and edges provide a general overview of the complexity of connections during the 0-back task and differences between the 3 groups. The Shirer atlas [31] had irregular regions; therefore estimates of the center of mass for each should be considered very approximate (S1 and S6 Tables). Networks of nodes were visualized using BrainNet Viewer [141]. Montreal Neurological Institute [140] coordinates show x (positive to right), y (positive anterior), z (positive superior), and anatomical left side on the left of each figure. Edges (springs) that were shared by all 3 groups were shown as black lines on the anatomical mesh diagrams. Nodes (balls) were colored for basal ganglia (BG, black), anterior salience (SA, red), posterior salience (SP, magenta), dorsal attention network (DAN, yellow), left executive control network (LE, lime), right executive control network (RE, dark green), dorsal default mode network (DD, cyan), precuneus network (PD, blue), and ventral default mode network (VD, teal).
(DOCX)

S2 Fig Nodes and edges shared by pairs of groups. Edges with d>1.6 and FDR<0.01 that were shared by SC & START (16 edges in 5 communities) (a, b) (S7 Table), SC & STOPP (4 edges in 3 communities) (c, d) (S8 Table), and START & STOPP (16 edges in 5 communities) (e, f) (S9 Table) generally connected frontal to parietal regions, or homoptic regions of the left and right cerebrum. Ball and spring models of nodes and edges are described in the legend for S1 Fig.
(DOCX)

S3 Fig Unique connectivity patterns for each group. Edges that were significant exclusively in SC (11 edges, 4 communities) (a, b) (S3 Table), START (16 edges 3 communities) (c, d) (S4 Table), and STOPP (19 edges, 6 communities) (e, f) (S5 Table) were depicted on anatomical and connectivity maps by thick lines (d>1.6 and FDR<0.01). Ball and spring models of nodes and edges are described in the legend for S1 Fig. Colored nodes had high betweenness connectivity or functional importance that was discussed in the text and Table 3.
(DOCX)
