# Supplementary material for: Connectivity differences between Gulf War Illness (GWI) phenotypes during a test of attention
Source: PLoS One. 2019 Dec 31;14(12):e0226481. doi: 10.1371/journal.pone.0226481 (PMC6938369; doi:10.1371/journal.pone.0226481)
Supplement: S1 Table — The anatomical regions that were activated synchronously as networks were shown with their original network classification [31] and the abbreviations used here (ROI). Corresponding Brodman Areas ({BA}) and approximately aligned BrainMap Intrinsic Connectivity Networks (ICN) [94] are given for further reference. ROIs were numbered for each network (reproduced with permission from Oxford University Press). Networks were listed as basal ganglia (BG), anterior (SA) and posterior salience (SP), Dorsal Attention Network (DAN), left and right executive control (LE, RE), and precuneus (PD), dorsal (DD), and ventral (VD) default mode networks. ROIs in the Shirer networks were large and irregularly shaped making it difficult to estimate Montreal Neurological Institute coordinates [140] that would capture the center of mass for each region. These coordinates were displayed as ball and spring plots [141] in S1–S3 Figs to depict approximate network connections. (DOCX) [file pone.0226481.s001.docx]

S1 Table. Reference brain regions of interest (ROIs) and networks. The anatomical regions that were activated synchronously as networks were shown with their original network classification [31] and the abbreviations used here (ROI). Corresponding Brodman Areas ({BA}) and approximately aligned BrainMap Intrinsic Connectivity Networks (ICN) [91] are given for further reference. ROIs were numbered for each network (reproduced with permission from Oxford University Press). Networks were listed as basal ganglia (BG), anterior (SA) and posterior salience (SP), Dorsal Attention Network (DAN), left and right executive control (LE, RE), and precuneus (PD), dorsal (DD), and ventral (VD) default mode networks. ROIs in the Shirer networks were large and irregularly shaped making it difficult to estimate Montreal Neurological Institute coordinates [138] that would capture the center of mass for each region. These coordinates were displayed as ball and spring plots in figures S1-S3 to depict approximate network connections.

| Network Classification {BA} (L on left) | ROI | Estimated MNI Coordinates | BrainMap ICN {BA} |
| --- | --- | --- | --- |
| Basal Ganglia |  |  |  |
| 1. Left caudate and thalamus | BG1 | -14,9,4 | 3 |
| 2. Right caudate, putamen and thalamus | BG2 | 14,9,4 | 3 |
| 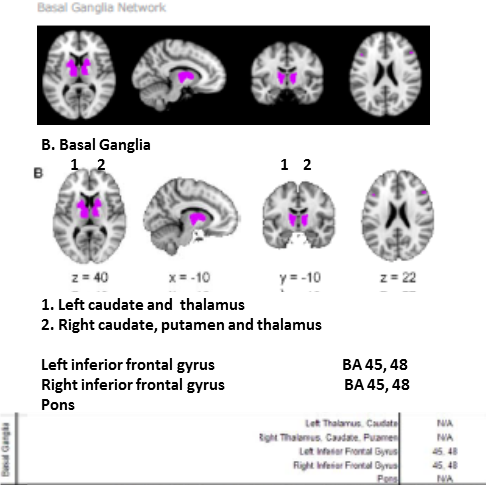 |  |  |  |
| Anterior Salience Network: Anterior Insula / Dorsal Anterior Cingulate Cortex (dACC) |  |  |  |
| 1. Left middle frontal gyrus {9,46} | SA1 | -47,31,23 | 7 {9,46} |
| 2. Left anterior insula {48,47} | SA2 | -34,28,-13 | 4 {48,47} |
| 3. Anterior cingulate cortex {24,32}, medial prefrontal cortex {8}, supplementary motor area {6} | SA3 | 0,11,41 | 4 {24} 6{8,6} 6{24,32} 7 {8} |
| 4. Right middle frontal gyrus {46,9} | SA4 | 43,33,17 | 7 {46,9} |
| 5. Right anterior insula {48,47} | SA5 | 38,30,-8 | 4 {48,47} |
| 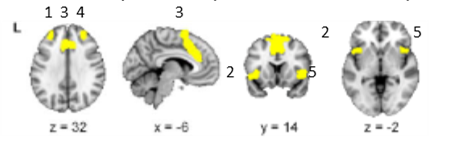 |  |  |  |
| Posterior Salience Network (Posterior Insula) |  |  |  |
| 1. Left supramarginal gyrus {40}, inferior parietal gyrus | SP1 | -53,-30,23 | 18 {40} |
| 2. Right supramarginal gyrus {2,40}, inferior parietal gyrus | SP2 | 56,-32,26 | 8 {2} 15 {40} |
| 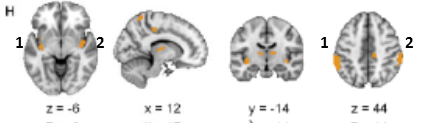 |  |  |  |
| DAN / Visuospatial Network (Intraparietal Sulcus & Frontal Eye Fields) (IPS & FEF) |  |  |  |
| 1. Left middle frontal gyrus, superior frontal gyrus, precentral gyrus (FEF) {6} | DAN1 | -23,-9,61 | 6 {6} |
| 2. Left inferior parietal sulcus {2,40,7} | DAN2 | -31,-56,38 | 8 {2} 7 {7} 15 {40} |
| 3. Right middle frontal gyrus {6} | DAN3 | 29,6,60 | 6 {6} |
| 4. Right inferior parietal lobule {2,40,7} | DAN4 | 27,-60,59 | 8 {2} 7 {7} 15 {40} |
| 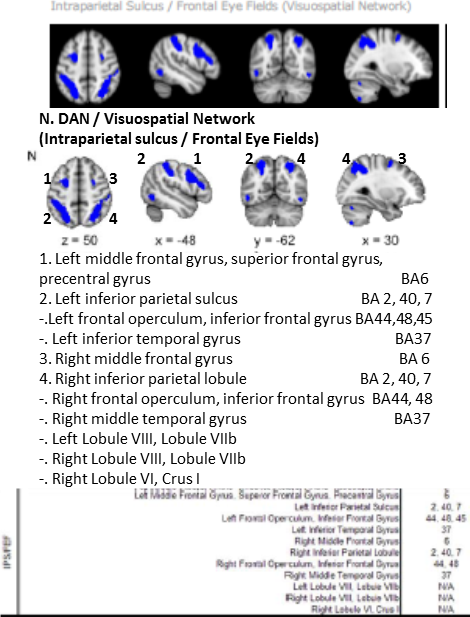 |  |  |  |
| Left Executive Control Network (L Dorsolateral Prefrontal Cortex / L Parietal) (DLPFC) |  |  |  |
| 1. Left middle frontal gyrus, superior frontal gyrus {8,9} | LE1 | -29,30,49 | 6 {8,9} |
| 2. Left inferior frontal gyrus {10,45}, orbitofrontal gyrus {47} | LE2 | -45,42,-3 | 18 {45 Pars} |
| 3. Left superior parietal gyrus {7}, inferior parietal gyrus {40}, precuneus, angular gyrus {39} | LE3 | -50,-44,-48 | 7 {7}  18 {40,39} |
| 4. Left inferior temporal gyrus, middle temporal gyrus {20,37} | LE4 | -49,-35,-14 | 10 {37} |
| 5. Right Crus 1 (cerebellum) | LE5 | 39,-60,-40 | 14 {Cbllm} |
| 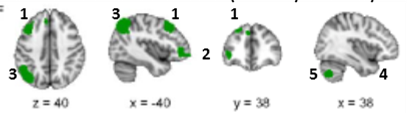 |  |  |  |
| Right Executive Control Network (R DLPFC / R Parietal) |  |  |  |
| 1. Right middle frontal gyrus, superior frontal gyrus {46,8,9} | RE1 | 40,28,43 | 7 {46,8,9} |
| 2. Right middle frontal gyrus {10,46} | RE2 | 48,49,7 | 7 {46} |
| 3. Right inferior parietal gyrus, supramarginal gyrus, angular gyrus {7,40,39} | RE3 | 48,-46,46 | 7 {7} 10 {39} 15 {40} |
| 4. Right superior frontal gyrus {8} | RE4 | 12,36,53 | 7 {8} |
| 5. Left Crus I, Crus II, Lobule VI (cerebellum) | RE5 | -38,-58,-32 | 14 {Cbllm} |
| 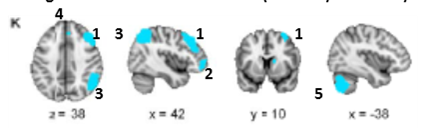 |  |  |  |
| Precuneus Default Mode Network |  |  |  |
| 1. Midcingulate cortex, posterior cingulate cortex {23} | PD1 | 0,-28,34 |  |
| 2. Precuneus, posterior {7,19} | PD2 | 0,-65,46 | 7 {7} |
| 3. Left angular gyrus {7,40} | PD3 | -39,-48,47 | 7 {7} |
| 4. Right angular gyrus {7,40} | PD4 | 38,-47,47 | 7 {7} 15 {40} |
| 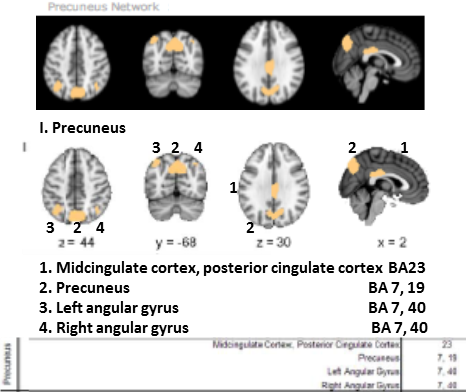 |  |  |  |
| Dorsal DMN (Posterior Cingulate Cortex / Medial Prefrontal Cortex) (PCC / MPFC) |  |  |  |
| 1. Medial prefrontal cortex, anterior cingulate cortex, orbitofrontal cortex; right superior frontal gyrus {9,10,24,32,11} | DD1 | 0,45,-5 | 6 {9} 4 {24} 2 {10,11} |
| 2. Left angular gyrus {39} | DD2 | -54,-57,33 | 10 {39} |
| 3. Posterior cingulate cortex (PCC), precuneus {23,30} | DD3 | 0,-45,20 | 1 {30} |
| 4. Right angular gyrus {39} | DD4 | 57,-51,32 | 10 {39} |
| 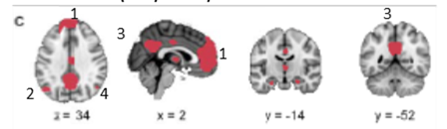 |  |  |  |
| Ventral Default Mode Network (Retrosplenial Cortex / Middle Temporal Lobe) |  |  |  |
| 1. Left retrospenial cortex, posterior cingulate {29,30,23} | VD1 | -7,-45,16 | 1 {30} |
| 2. Left middle frontal gyrus {8,6} | VD2 | 31,13,56 | 6 {8,6} |
| 3. Left parahippocampal gyrus {37,20} | VD3 | -53,-39,-26 | 1 {37,20} |
| 4. Left middle occipital gyrus {19,39} | VD4 | -53,-66,11 | 11-13 {19} 10 {39} |
| 5. Right retrospenial & posterior cingulate cortex {30,23} | VD5 | 7,-44,14 | 1 {30} |
| 6. Precuneus {5,7} | VD6 | 0,-47,75 | 9 {5} 7 {7} |
| 7. Right superior frontal gyrus, middle frontal gyrus {9,8} | VD7 | 24,39,37 | 6 {9,8} |
| 8. Right parahippocampal gyrus {37, 30} | VD8 | 61,-42,-25 | 1 {37,30} |
| 9. Right angular gyrus, middle occipital gyrus {39, 19} | VD9 | 60,-61,8 | 1 {39} 11-13 {19} |
| 10. Right lobule II (cerebellum) | VD10 | 10,-49,-29 | 14 {Cbllm} |
| 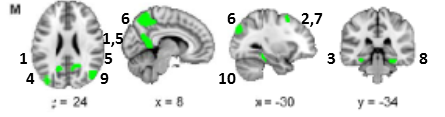 |  |  |  |
