# Supplementary material for: Connectivity differences between Gulf War Illness (GWI) phenotypes during a test of attention
Source: PLoS One. 2019 Dec 31;14(12):e0226481. doi: 10.1371/journal.pone.0226481 (PMC6938369; doi:10.1371/journal.pone.0226481)
Supplement: S2 Table — (DOCX) [file pone.0226481.s002.docx]

Table S2. Timing for 0-back and 2-back paradigm in ePrime software [61].

| Block Repetition | Condition | View on screen and task | Duration (sec) | Time at start | Time at end |
| --- | --- | --- | --- | --- | --- |
| 0 | Dummy | Blank screen | 6 | 0 | 6 |
| 1 | Instruction | "0-BACK" | 0.8 | 6 | 6.8 |
| 1 | Blank | Blank screen | 1.2 | 6.8 | 8 |
| 1 | 0-back | sequence of 9 letters x 2 sec each, press button 9 times | 18 | 8 | 26 |
| 1 | Instruction | "REST" | 0.8 | 26 | 26.8 |
| 1 | Blank | Blank screen | 1.2 | 26.8 | 28 |
| 1 | Fixation | Cross hair fixation | 8 | 28 | 36 |
| 1 | Instruction | "2-BACK" | 0.8 | 36 | 36.8 |
| 1 | Blank | Blank screen | 1.2 | 36.8 | 38 |
| 1 | 2-back | sequence of 9 letters x 2 sec each, press button 7 times | 18 | 38 | 56 |
| 1 | Instruction | "REST" | 0.8 | 56 | 56.8 |
| 1 | Blank | Blank screen | 1.2 | 56.8 | 58 |
| 1 | Fixation | Cross hair fixation | 8 | 58 | 66 |
| 2 | Instruction | "0-BACK" | 0.8 | 66 | 66.8 |
| 2 | Blank | Blank screen | 1.2 | 66.8 | 68 |
| 2 | 0-Back | sequence of 9 letters x 2 sec each, press button 9 times | 18 | 68 | 86 |
| 2 | Instruction | "REST" | 0.8 | 86 | 86.8 |
| 2 | Blank | Blank screen | 1.2 | 86.8 | 88 |
| 2 | Fixation | Cross hair fixation | 8 | 88 | 96 |
| 2 | Instruction | "2-BACK" | 0.8 | 96 | 96.8 |
| 2 | Blank | Blank screen | 1.2 | 96.8 | 98 |
| 2 | 2-back | sequence of 9 letters x 2 sec each, press button 7 times | 18 | 98 | 116 |
| 2 | Instruction | "REST" | 0.8 | 116 | 116.8 |
| 2 | Blank | Blank screen | 1.2 | 116.8 | 118 |
| 2 | Fixation | Cross hair fixation | 8 | 118 | 126 |
| 3 | Instruction | "0-BACK" | 0.8 | 126 | 126.8 |
| 3 | Blank | Blank screen | 1.2 | 126.8 | 128 |
| 3 | 0-Back | sequence of 9 letters x 2 sec each, press button 9 times | 18 | 128 | 146 |
| 3 | Instruction | "REST" | 0.8 | 146 | 146.8 |
| 3 | Blank | Blank screen | 1.2 | 146.8 | 148 |
| 3 | Fixation | Cross hair fixation | 8 | 148 | 156 |
| 3 | Instruction | "2-BACK" | 0.8 | 156 | 156.8 |
| 3 | Blank | Blank screen | 1.2 | 156.8 | 158 |
| 3 | 2-back | sequence of 9 letters x 2 sec each, press button 7 times | 18 | 158 | 176 |
| 3 | Instruction | "REST" | 0.8 | 176 | 176.8 |
| 3 | Blank | Blank screen | 1.2 | 176.8 | 178 |
| 3 | Fixation | Cross hair fixation | 8 | 178 | 186 |
| 4 | Instruction | "0-BACK" | 0.8 | 186 | 186.8 |
| 4 | Blank | Blank screen | 1.2 | 186.8 | 188 |
| 4 | 0-Back | sequence of 9 letters x 2 sec each, press button 9 times | 18 | 188 | 206 |
| 4 | Instruction | "REST" | 0.8 | 206 | 206.8 |
| 4 | Blank | Blank screen | 1.2 | 206.8 | 208 |
| 4 | Fixation | Cross hair fixation | 8 | 208 | 216 |
| 4 | Instruction | "2-BACK" | 0.8 | 216 | 216.8 |
| 4 | Blank | Blank screen | 1.2 | 216.8 | 218 |
| 4 | 2-Back | sequence of 9 letters x 2 sec each, press button 7 times | 18 | 218 | 236 |
| 4 | Instruction | "REST" | 0.8 | 236 | 236.8 |
| 4 | Blank | Blank screen | 1.2 | 236.8 | 238 |
| 4 | Fixation | Cross hair fixation | 8 | 238 | 246 |
| 5 | Instruction | "0-BACK" | 0.8 | 246 | 246.8 |
| 5 | Blank | Blank screen | 1.2 | 246.8 | 248 |
| 5 | 0-Back | sequence of 9 letters x 2 sec each, press button 9 times | 18 | 248 | 266 |
| 5 | Instruction | "REST" | 0.8 | 266 | 266.8 |
| 5 | Blank | Blank screen | 1.2 | 266.8 | 268 |
| 5 | Fixation | Cross hair fixation | 8 | 268 | 276 |
| 5 | Instruction | "2-BACK" | 0.8 | 276 | 276.8 |
| 5 | Blank | Blank screen | 1.2 | 276.8 | 278 |
| 5 | 2-Back | sequence of 9 letters x 2 sec each, press button 7 times | 18 | 278 | 296 |
| 5 | Instruction | "REST" | 0.8 | 296 | 296.8 |
| 5 | Blank | Blank screen | 1.2 | 296.8 | 298 |
| 5 | Fixation | Cross hair fixation | 8 | 298 | 306 |
