# Supplementary material for: Connectivity differences between Gulf War Illness (GWI) phenotypes during a test of attention
Source: PLoS One. 2019 Dec 31;14(12):e0226481. doi: 10.1371/journal.pone.0226481 (PMC6938369; doi:10.1371/journal.pone.0226481)
Supplement: S3 Table — All significant edges in individual groups, pairs of groups, and the entire group were tabulated with the average Fisher’s z-transformed Pearson’s correlation coefficients, standard deviations, Cohen’s d (d > 1.6), and Student’s t-test (FDR < 0.01). Edges were arranged by connected modules (S1 Fig). The anatomical location, estimated approximate Montreal Neurological Institute (MNI) coordinates from the original reference [31], and most closely aligned BrainMap Intrinsic Connectivity Network (ICN) [94] were shown for each node. (DOCX) [file pone.0226481.s003.docx]

Table S3. Nodes and edges shared by the SC, START and STOPP groups. All significant edges in individual groups, pairs of groups, and the entire group were tabulated with the average Fisher’s z-transformed Pearson’s correlation coefficients, standard deviations, Cohen’s d (d > 1.6), and Student’s t-test (FDR < 0.01). Edges were arranged by connected modules (Figure S1). The anatomical location, estimated approximate Montreal Neurological Institute (MNI) coordinates from the original reference [30], and most closely aligned BrainMap Intrinsic Connectivity Network (ICN) [90] were shown for each node.

| Group | Node 1 | Node 2 | Avg | SD | d | FDR | Node 1 Anatomy {BA} | Node 1 MNI | Brain Map20 ICN {BA} | Node 2 Anatomy {BA} | Node 2 MNI | Brain Map20 ICN {BA} |
| --- | --- | --- | --- | --- | --- | --- | --- | --- | --- | --- | --- | --- |
| 0-back core task (right dorsolateral prefrontal cortex) network | | | | | | | | | | | | |
| SC, START & STOPP | RE1 | RE4 | 0.89 | 0.42 | 1.65 | 0.0050 | Right middle frontal gyrus, superior frontal gyrus {46,8,9} | 40,28,43 | 7 {46,8,9} | Right superior frontal gyrus {8} | 12,36,53 | 7 {8} |
| SC, START & STOPP | RE1 | VD7 | 1.20 | 0.37 | 1.79 | 0.00070 | Right middle frontal gyrus, superior frontal gyrus {46,8,9} | 40,28,43 | 7 {46,8,9} | Right superior frontal gyrus, middle frontal gyrus {9,8} | 24,39,37 | 6 {9,8} |
| SC, START & STOPP | RE4 | VD7 | 0.92 | 0.45 | 1.63 | 0.0060 | Right superior frontal gyrus {8} | 12,36,53 | 7 {8} | Right superior frontal gyrus, middle frontal gyrus {9,8} | 24,39,37 | 6 {9,8} |
| 0-back core dorsal attention network (DAN) | | | | | | | | | | | | |
| SC, START & STOPP | DAN1 | SP1 | 0.88 | 0.38 | 1.68 | 0.0034 | Left middle frontal gyrus, superior frontal gyrus, Precentral gyrus (FEF) {6} | -23,-9,61 | 6 {6} | Left supramarginal gyrus, inferior parietal gyrus {40} | -53,-30,23 | 18 {40} |
| SC, START & STOPP | DAN1 | DAN3 | 1.14 | 0.40 | 1.76 | 0.0012 | Left middle frontal gyrus, superior frontal gyrus, Precentral gyrus (FEF) {6} | -23,-9,61 | 6 {6} | Right middle frontal gyrus (FEF) {6} | 29,6,60 | 6 {6} |
| SC, START & STOPP | DAN3 | PD4 | 0.72 | 0.32 | 1.66 | 0.0041 | Right middle frontal gyrus (FEF) {6} | 29,6,60 | 6 {6} | Right angular gyrus {7,40} supramarginal gyrus, superior parietal cortex | 38,-47,47 | 7 {7} 15 {40} |
| SC, START & STOPP | RE3 | PD4 | 1.32 | 0.53 | 1.71 | 0.0025 | Right inferior parietal gyrus, supramarginal gyrus, angular gyrus {7,40,39} | 48,-46,46 | 7 {7} 10 {39} 15 {40} | Right angular gyrus {7,40} supramarginal gyrus, superior parietal cortex | 38,-47,47 | 7 {7} 15 {40} |
| 0-back core default mode network | | | | | | | | | | | | |
| SC, START & STOPP | VD1 | VD5 | 1.12 | 0.27 | 1.85 | 0.00020 | Left retrospenial cortex, posterior cingulate {29,30,23} | -7,-45,16 | 1 {30} | Right retrospenial & posterior cingulate cortex {30,23} | 7,-44,14 | 1 {30} |
| SC, START & STOPP | DD3 | VD5 | 0.90 | 0.25 | 1.81 | 0.00046 | Posterior cingulate cortex (PCC), precuneus (inferior) {23,30} | 0,-45,20 | 1 {30} | Right retrospenial & posterior cingulate cortex {30,23} | 7,-44,14 | 1 {30} |
| SC, START & STOPP | DD3 | VD1 | 0.99 | 0.25 | 1.84 | 0.00027 | Posterior cingulate cortex (PCC), precuneus (inferior) {23,30} | 0,-45,20 | 1 {30} | Left retrospenial cortex, posterior cingulate {29,30,23} | -7,-45,16 | 1 {30} |
| SC, START & STOPP | DD2 | DD3 | 0.80 | 0.38 | 1.64 | 0.0053 | Left angular gyrus {39} | -54,-57,33 | 10 {39} | Posterior cingulate cortex (PCC), precuneus (inferior) {23,30} | 0,-45,20 | 1 {30} |
| SC, START & STOPP | RE2 | DD2 | 1.05 | 0.27 | 1.83 | 0.00028 | Right middle frontal gyrus {10,46} | 48,49,7 | 7 {46} | Left angular gyrus {39} | -54,-57,33 | 10 {39} |
| SC, START & STOPP | DD2 | VD4 | 0.78 | 0.37 | 1.63 | 0.00556 | Left angular gyrus {39} | -54,-57,33 | 10 {39} | Left middle occipital gyrus {19,39} | -53,-66,11 | 11-13 {19} 10 {39} |
| SC, START & STOPP | VD4 | VD6 | 0.71 | 0.34 | 1.64 | 0.0055 | Left middle occipital gyrus {19,39} | -53,-66,11 | 11-13 {19} 10 {39} | Precuneus (superior) {5,7} | 0,-47,75 | 9 {5} 7 {7} |
| SC, START & STOPP | LE3 | VD4 | 0.79 | 0.41 | 1.60 | 0.0072 | Left superior parietal gyrus {7}, inferior parietal gyrus {40}, precuneus, angular gyrus {39} | -50,-44,-48 | 7 {7} 18 {40,39} | Left middle occipital gyrus {19,39} | -53,-66,11 | 11-13 {19} 10 {39} |
| SC, START & STOPP | PD2 | VD6 | 0.91 | 0.40 | 1.68 | 0.0036 | Precuneus (posterior) {7,19} | 0,-65,46 | 7 {7} | Precuneus (superior) {5,7} | 0,-47,75 | 9 {5} 7 {7} |
| SC, START & STOPP | LE3 | PD2 | 0.82 | 0.32 | 1.73 | 0.0020 | Left superior parietal gyrus {7}, inferior parietal gyrus {40}, precuneus, angular gyrus {39} | -50,-44,-48 | 7 {7} 18 {40,39} | Precuneus (posterior) {7,19} | 0,-65,46 | 7 {7} |
| SC, START & STOPP | LE3 | PD3 | 1.77 | 0.48 | 1.82 | 0.00036 | Left superior parietal gyrus {7}, inferior parietal gyrus {40}, precuneus, angular gyrus {39} | -50,-44,-48 | 7 {7} 18 {40,39} | Left angular gyrus {7,40} supramarginal gyrus, superior parietal cortex | -39,-48,47 | 7 {7} |
| Pairs of nodes | | | | | | | | | | | | |
| SC, START & STOPP | BG1 | BG2 | 1.12 | 0.32 | 1.81 | 0.00046 | Left caudate and thalamus | -14,9,4 | 3 | Right caudate, putamen and thalamus | 14,9,4 | 3 |
| SC, START & STOPP | SA2 | SA5 | 0.80 | 0.36 | 1.66 | 0.0042 | Left anterior insula {48,47} | -34,28,-13 | 4 {48,47} | Right anterior insula {48,47} | 38,30,-8 | 4 {48,47} |
| SC, START & STOPP | LE1 | VD2 | 0.96 | 0.29 | 1.80 | 0.00063 | Left middle frontal gyrus, superior frontal gyrus (SMA, PMC {8}, DLPFC {9}) | -29,30,49 | 6 {8,9} | Left middle frontal gyrus {8,6} | 31,13,56 | 6 {8,6} |
