# Supplementary material for: Connectivity differences between Gulf War Illness (GWI) phenotypes during a test of attention
Source: PLoS One. 2019 Dec 31;14(12):e0226481. doi: 10.1371/journal.pone.0226481 (PMC6938369; doi:10.1371/journal.pone.0226481)
Supplement: S4 Table — All significant edges in individual groups, pairs of groups, and the entire group were tabulated with the average Fisher’s z-transformed Pearson’s correlation coefficients, standard deviations, Cohen’s d (d > 1.6), and Student’s t-test (FDR < 0.01). Edges were arranged by connected modules (S2 Fig). The anatomical location, estimated approximate Montreal Neurological Institute (MNI) coordinates from the original reference [31], and most closely aligned BrainMap Intrinsic Connectivity Network (ICN) [94] were shown for each node. (DOCX) [file pone.0226481.s004.docx]

Table S4. Nodes and edges shared by the SC and STOPP groups. All significant edges in individual groups, pairs of groups, and the entire group were tabulated with the average Fisher’s z-transformed Pearson’s correlation coefficients, standard deviations, Cohen’s d (d > 1.6), and Student’s t-test (FDR < 0.01). Edges were arranged by connected modules (Fig S2). The anatomical location, estimated approximate Montreal Neurological Institute (MNI) coordinates from the original reference [30], and most closely aligned BrainMap Intrinsic Connectivity Network (ICN) [90] were shown for each node.

| Group | Node 1 | Node 2 | Avg | SD | d | FDR | Node 1 Anatomy {BA} | Node 1 MNI | Brain Map20 ICN {BA} | Node 2 Anatomy {BA} | Node 2 MNI | Brain Map20 ICN {BA} |
| --- | --- | --- | --- | --- | --- | --- | --- | --- | --- | --- | --- | --- |
| Chain of task nodes | | | | | | | | | | | | |
| SC & STOPP | SA4 | VD7 | 0.87 | 0.31 | 1.77 | 3.5E-7 | Right middle frontal gyrus {46,9} | 43,33,17 | 7 {46,9} | Right superior frontal gyrus, middle frontal gyrus {9,8} | 24,39,37 | 6 {9,8} |
| SC & STOPP | SA4 | RE1 | 0.87 | 0.36 | 1.72 | 1.4E-6 | Right middle frontal gyrus {46,9} | 43,33,17 | 7 {46,9} | Right middle frontal gyrus, superior frontal gyrus {46,8,9} | 40,28,43 | 7 {46,8,9} |
| SC & STOPP | SA3 | SA4 | 0.71 | 0.25 | 1.78 | 2.7E-7 | Anterior cingulate cortex {24,32}, medial prefrontal cortex {8}, supplementary motor area {6} | 0,11,41 | 4 {24} 6{8,6} 6{24,32} 7 {8} | Right middle frontal gyrus {46,9} | 43,33,17 | 7 {46,9} |
| SC & STOPP | SA3 | VD2 | 0.71 | 0.33 | 1.67 | 4.4E-6 | Anterior cingulate cortex {24,32}, medial prefrontal cortex {8}, supplementary motor area {6} | 0,11,41 | 4 {24} 6{8,6} 6{24,32} 7 {8} | Left middle frontal gyrus {8,6} | 31,13,56 | 6 {8,6} |
| Right frontal eye field chain | | | | | | | | | | | | |
| SC & STOPP | DAN3 | SP2 | 0.73 | 0.28 | 1.75 | 5.3E-7 | Right middle frontal gyrus (FEF) {6} | 29,6,60 | 6 {6} | Right supramarginal gyrus, inferior parietal gyrus {2,40} | 56,-32,26 | 8 {2} 15 {40} |
| SC & STOPP | DAN3 | VD6 | 0.76 | 0.37 | 1.64 | 8.8E-6 | Right middle frontal gyrus (FEF) {6} | 29,6,60 | 6 {6} | Precuneus (superior) {5,7} | 0,-47,75 | 9 {5} 7 {7} |
| Default network centered on posterior precuneus (PD2) | | | | | | | | | | | | |
| SC & STOPP | PD3 | DD2 | 0.67 | 0.30 | 1.68 | 3.7E-6 | Left angular gyrus {7,40} supramarginal gyrus, superior parietal cortex | -39,-48,47 | 7 {7} | Left angular gyrus {39} | -54,-57,33 | 10 {39} |
| SC & STOPP | RE3 | PD3 | 0.75 | 0.29 | 1.75 | 6.2E-7 | Right inferior parietal gyrus, supramarginal gyrus, angular gyrus {7,40,39} | 48,-46,46 | 7 {7} 10 {39} 15 {40} | Left angular gyrus {7,40} supramarginal gyrus, superior parietal cortex | -39,-48,47 | 7 {7} |
| SC & STOPP | PD3 | PD4 | 0.70 | 0.24 | 1.78 | 2.5E-7 | Left angular gyrus {7,40} supramarginal gyrus, superior parietal cortex | -39,-48,47 | 7 {7} | Right angular gyrus {7,40} supramarginal gyrus, superior parietal cortex | 38,-47,47 | 7 {7} 15 {40} |
| SC & STOPP | LE3 | PD4 | 0.68 | 0.29 | 1.70 | 2.0E-6 | Left superior parietal gyrus {7}, inferior parietal gyrus {40}, precuneus, angular gyrus {39} | -50,-44,-48 | 7 {7} 18 {40,39} | Right angular gyrus {7,40} supramarginal gyrus, superior parietal cortex | 38,-47,47 | 7 {7} 15 {40} |
| SC & STOPP | PD2 | PD3 | 0.76 | 0.36 | 1.65 | 6.6E-6 | Precuneus (posterior) {7,19} | 0,-65,46 | 7 {7} | Left angular gyrus {7,40} supramarginal gyrus, superior parietal cortex | -39,-48,47 | 7 {7} |
| SC & STOPP | PD2 | PD4 | 0.59 | 0.29 | 1.64 | 9.2E-6 | Precuneus (posterior) {7,19} | 0,-65,46 | 7 {7} | Right angular gyrus {7,40} supramarginal gyrus, superior parietal cortex | 38,-47,47 | 7 {7} 15 {40} |
| SC & STOPP | RE3 | PD2 | 0.57 | 0.22 | 1.74 | 7.2E-7 | Right inferior parietal gyrus, supramarginal gyrus, angular gyrus {7,40,39} | 48,-46,46 | 7 {7} 10 {39} 15 {40} | Precuneus (posterior) {7,19} | 0,-65,46 | 7 {7} |
| SC & STOPP | LE3 | RE3 | 0.80 | 0.34 | 1.71 | 1.9E-6 | Left superior parietal gyrus {7}, inferior parietal gyrus {40}, precuneus, angular gyrus {39} | -50,-44,-48 | 7 {7} 18 {40,39} | Right inferior parietal gyrus, supramarginal gyrus, angular gyrus {7,40,39} | 48,-46,46 | 7 {7} 10 {39} 15 {40} |
| 0-back core default mode network | | | | | | | | | | | | |
| SC & STOPP | PD1 | DD3 | 0.56 | 0.29 | 1.60 | 1.8E-5 | Midcingulate cortex, posterior cingulate cortex {23} | 0,-28,34 |  | Posterior cingulate cortex (PCC), precuneus (inferior) {23,30} | 0,-45,20 | 1 {30} |
| Parahippocampal edge | | | | | | | | | | | | |
| SC & STOPP | VD3 | VD8 | 0.53 | 0.26 | 1.64 | 8.5E-6 | Left parahippocampal gyrus {37,20} | -53,-39,-26 | 1 {37,20} | Right parahippocampal gyrus {37,30} | 61,-42,-25 | 1 {37,30} |
