# Supplementary material for: Connectivity differences between Gulf War Illness (GWI) phenotypes during a test of attention
Source: PLoS One. 2019 Dec 31;14(12):e0226481. doi: 10.1371/journal.pone.0226481 (PMC6938369; doi:10.1371/journal.pone.0226481)
Supplement: S5 Table — All significant edges in individual groups, pairs of groups, and the entire group were tabulated with the average Fisher’s z-transformed Pearson’s correlation coefficients, standard deviations, Cohen’s d (d > 1.6), and Student’s t-test (FDR < 0.01). Edges were arranged by connected modules (S2 Fig). The anatomical location, estimated approximate Montreal Neurological Institute (MNI) coordinates from the original reference [31], and most closely aligned BrainMap Intrinsic Connectivity Network (ICN) [94] were shown for each node. (DOCX) [file pone.0226481.s005.docx]

Table S5. Nodes and edges shared by the START and STOPP groups. All significant edges in individual groups, pairs of groups, and the entire group were tabulated with the average Fisher’s z-transformed Pearson’s correlation coefficients, standard deviations, Cohen’s d (d > 1.6), and Student’s t-test (FDR < 0.01). Edges were arranged by connected modules (Fig S2). The anatomical location, estimated approximate Montreal Neurological Institute (MNI) coordinates from the original reference [30], and most closely aligned BrainMap Intrinsic Connectivity Network (ICN) [90] were shown for each node.

| Group | Node 1 | Node 2 | Avg | SD | d | FDR | Node 1 Anatomy {BA} | Node 1 MNI | Brain Map20 ICN {BA} | Node 2 Anatomy {BA} | Node 2 MNI | Brain Map20 ICN {BA} | |
| --- | --- | --- | --- | --- | --- | --- | --- | --- | --- | --- | --- | --- | --- |
| Parietal dorsal attention network edge | | | | | | | | | | | | | |
| START & STOPP | DAN2 | DAN4 | 0.56 | 0.27 | 1.65 | 0.0028 | Left inferior parietal sulcus {2,40,7} | -31,-56,38 | 8 {2} 7 {7} 15 {40} | Right inferior parietal lobule {2,40,7} | 27,-60,59 | 8 {2} 7 {7} 15 {40} | |
| Dorsal anterior cingulate cortex – dorsolateral prefrontal cortex task network | | | | | | | | | | | | | |
| START & STOPP | RE1 | RE2 | 0.85 | 0.27 | 1.81 | 0.00028 | Right middle frontal gyrus, superior frontal gyrus {46,8,9} | 40,28,43 | 7 {46,8,9} | Right middle frontal gyrus {10,46} | 48,49,7 | 7 {46} | |
| START & STOPP | SA3 | RE1 | 0.62 | 0.20 | 1.80 | 0.00033 | Anterior cingulate cortex {24,32}, medial prefrontal cortex {8}, supplementary motor area {6} | 0,11,41 | 4 {24} 6{8,6} 6{24,32} 7 {8} | Right middle frontal gyrus, superior frontal gyrus {46,8,9} | 40,28,43 | 7 {46,8,9} | |
| START & STOPP | SA3 | RE4 | 0.65 | 0.27 | 1.71 | 0.0014 | Anterior cingulate cortex {24,32}, medial prefrontal cortex {8}, supplementary motor area {6} | 0,11,41 | 4 {24} 6{8,6} 6{24,32} 7 {8} | Right superior frontal gyrus {8} | 12,36,53 | 7 {8} | |
| START & STOPP | SA3 | VD7 | 0.73 | 0.19 | 1.85 | 9.3E-5 | Anterior cingulate cortex {24,32}, medial prefrontal cortex {8}, supplementary motor area {6} | 0,11,41 | 4 {24} 6{8,6} 6{24,32} 7 {8} | Right superior frontal gyrus, middle frontal gyrus {9,8} | 24,39,37 | 6 {9,8} | |
| Left angular gyrus default chain | | | | | | | | | | | | |  |
| START & STOPP | DD2 | VD1 | 0.52 | 0.27 | 1.62 | 0.0041 | Left angular gyrus {39} | -54,-57,33 | 10 {39} | Left retrosplenial cortex, posterior cingulate {29,30,23} | -7,-45,16 | 1 {30} | |
| START & STOPP | DD2 | VD5 | 0.37 | 0.20 | 1.61 | 0.0046 | Left angular gyrus {39} | -54,-57,33 | 10 {39} | Right retrosplenial & posterior cingulate cortex {30,23} | 7,-44,14 | 1 {30} | |
| Precuneus centered default network | | | | | | | | | | | | |  |
| START & STOPP | LE2 | DD4 | 0.67 | 0.30 | 1.69 | 0.0019 | Left inferior frontal gyrus {10,45}, orbitofrontal gyrus {47} | -45,42,-3 | 18 {45, Pars} | Right angular gyrus {39} | 57,-51,32 | 10 {39} | |
| START & STOPP | DD4 | VD9 | 0.73 | 0.33 | 1.68 | 0.0020 | Right angular gyrus {39} | 57,-51,32 | 10 {39} | Right angular gyrus, middle occipital gyrus {39,19} | 60,-61,8 | 1 {39} 11-13 {19} | |
| START & STOPP | PD4 | VD9 | 0.80 | 0.38 | 1.65 | 0.0030 | Right angular gyrus {7,40} supramarginal gyrus, superior parietal cortex | 38,-47,47 | 7 {7} 15 {40} | Right angular gyrus, middle occipital gyrus {39,19} | 60,-61,8 | 1 {39} 11-13 {19} | |
| START & STOPP | PD4 | VD6 | 0.63 | 0.29 | 1.67 | 0.0023 | Right angular gyrus {7,40} supramarginal gyrus, superior parietal cortex | 38,-47,47 | 7 {7} 15 {40} | Precuneus (superior) {5,7} | 0,-47,75 | 9 {5} 7 {7} | |
| START & STOPP | PD3 | VD6 | 0.67 | 0.25 | 1.75 | 0.00080 | Left angular gyrus {7,40} supramarginal gyrus, superior parietal cortex | -39,-48,47 | 7 {7} | Precuneus (superior) {5,7} | 0,-47,75 | 9 {5} 7 {7} | |
| START & STOPP | SP1 | VD6 | 0.64 | 0.28 | 1.70 | 0.0016 | Left supramarginal gyrus, inferior parietal gyrus {40} | -53,-30,23 | 18 {40} | Precuneus (superior) {5,7} | 0,-47,75 | 9 {5} 7 {7} | |
| START & STOPP | LE3 | VD6 | 0.63 | 0.23 | 1.75 | 0.00075 | Left superior parietal gyrus {7}, inferior parietal gyrus {40}, precuneus, angular gyrus {39} | -50,-44,-48 | 7 {7} 18 {40,39} | Precuneus (superior) {5,7} | 0,-47,75 | 9 {5} 7 {7} | |
| Left middle occipital gyrus default chain | | | | | | | | | | | | |  |
| START & STOPP | DD3 | VD4 | 0.42 | 0.23 | 1.60 | 0.0047 | Posterior cingulate cortex (PCC), precuneus (inferior) {23,30} | 0,-45,20 | 1 {30} | Left middle occipital gyrus {19,39} | -53,-66,11 | 11-13 {19} 10 {39} | |
| START & STOPP | PD2 | VD4 | 0.76 | 0.28 | 1.76 | 0.00070 | Precuneus (posterior) {7,19} | 0,-65,46 | 7 {7} | Left middle occipital gyrus {19,39} | -53,-66,11 | 11-13 {19} 10 {39} | |
