# Supplementary material for: Connectivity differences between Gulf War Illness (GWI) phenotypes during a test of attention
Source: PLoS One. 2019 Dec 31;14(12):e0226481. doi: 10.1371/journal.pone.0226481 (PMC6938369; doi:10.1371/journal.pone.0226481)
Supplement: S6 Table — All significant edges in individual groups, pairs of groups, and the entire group were tabulated with the average Fisher’s z-transformed Pearson’s correlation coefficients, standard deviations, Cohen’s d (d > 1.6), and Student’s t-test (FDR < 0.01). Edges were arranged by connected modules (S2 Fig). The anatomical location, estimated approximate Montreal Neurological Institute (MNI) coordinates from the original reference [31], and most closely aligned BrainMap Intrinsic Connectivity Network (ICN) [94] were shown for each node. (DOCX) [file pone.0226481.s006.docx]

Table S6. Nodes and edges shared by the SC and START groups. All significant edges in individual groups, pairs of groups, and the entire group were tabulated with the average Fisher’s z-transformed Pearson’s correlation coefficients, standard deviations, Cohen’s d (d > 1.6), and Student’s t-test (FDR < 0.01). Edges were arranged by connected modules (Fig S2). The anatomical location, estimated approximate Montreal Neurological Institute (MNI) coordinates from the original reference [30], and most closely aligned BrainMap Intrinsic Connectivity Network (ICN) [90] were shown for each node.

| Group | Node 1 | Node 2 | Avg | SD | d | FDR | Node 1 Anatomy {BA} | Node 1 MNI | Brain Map20 ICN {BA} | Node 2 Anatomy {BA} | Node 2 MNI | Brain Map20 ICN {BA} |
| --- | --- | --- | --- | --- | --- | --- | --- | --- | --- | --- | --- | --- |
| Left frontal eye field – dorsal attention chain | | | | | | | | | | | | |
| SC & START | DAN1 | VD6 | 0.76 | 0.35 | 1.67 | 0.0024 | Left middle frontal gyrus, superior frontal gyrus, Precentral gyrus (FEF) {6} | -23,-9,61 | 6 {6} | Precuneus (superior) {5,7} | 0,-47,75 | 9 {5} 7 {7} |
| SC & START | DAN1 | PD3 | 0.66 | 0.34 | 1.62 | 0.0039 | Left middle frontal gyrus, superior frontal gyrus, Precentral gyrus (FEF) {6} | -23,-9,61 | 6 {6} | Left angular gyrus {7,40} supramarginal gyrus, superior parietal cortex | -39,-48,47 | 7 {7} |
| Individual edges in START | | | | | | | | | | | | |
| SC & START | VD4 | VD9 | 0.61 | 0.27 | 1.70 | 0.0016 | Left middle occipital gyrus {19,39} | -53,-66,11 | 11-13 {19} 10 {39} | Right angular gyrus, middle occipital gyrus {39,19} | 60,-61,8 | 1 {39} 11-13 {19} |
| SC & START | DAN3 | SP1 | 0.50 | 0.19 | 1.74 | 0.00094 | Right middle frontal gyrus (FEF) {6} | 29,6,60 | 6 {6} | Left supramarginal gyrus, inferior parietal gyrus {40} | -53,-30,23 | 18 {40} |
