# Supplementary material for: Connectivity differences between Gulf War Illness (GWI) phenotypes during a test of attention
Source: PLoS One. 2019 Dec 31;14(12):e0226481. doi: 10.1371/journal.pone.0226481 (PMC6938369; doi:10.1371/journal.pone.0226481)
Supplement: S7 Table — All significant edges in individual groups, pairs of groups, and the entire group were tabulated with the average Fisher’s z-transformed Pearson’s correlation coefficients, standard deviations, Cohen’s d (d > 1.6), and Student’s t-test (FDR < 0.01). Edges were arranged by system modules (S3 Fig). The anatomical location from Shirer et al. [31], approximated center of mass in Montreal Neurological Institute (MNI) coordinates [140], and most closely aligned BrainMap Intrinsic Connectivity Network (ICN) [94] were estimated for each node. (DOCX) [file pone.0226481.s007.docx]

Table S7. Nodes and edges in SC group. All significant edges in individual groups, pairs of groups, and the entire group were tabulated with the average Fisher’s z-transformed Pearson’s correlation coefficients, standard deviations, Cohen’s d (d > 1.6), and Student’s t-test (FDR < 0.01). Edges were arranged by system modules (Fig S3). The anatomical location from Shirer et al. [30], approximated center of mass in Montreal Neurological Institute (MNI) coordinates [138], and most closely aligned BrainMap Intrinsic Connectivity Network (ICN) [91] were estimated for each node.

| Group | Node 1 | Node 2 | Avg | SD | d | FDR | Node 1 Anatomy | Node 1 MNI | Brain Map20 ICN {BA} | Node 2 Anatomy | Node 2 MNI | Brain Map20 ICN {BA} |
| --- | --- | --- | --- | --- | --- | --- | --- | --- | --- | --- | --- | --- |
| Pairs of nodes, unconnected edges | | | | | | | | | | | | |
| SC | SA3 | SP2 | 0.50 | 0.23 | 1.65 | 0.0047 | Anterior cingulate cortex {24,32}, medial prefrontal cortex {8}, supplementary motor area {6} | 0,11,41 | 4 {24} 6{8,6} 6{24,32} 7 {8} | Right supramarginal gyrus, inferior parietal gyrus {2,40} | 56,-32,26 | 8 {2} 15 {40} |
| SC | SP1 | VD2 | 0.40 | 0.20 | 1.62 | 0.0063 | Left supramarginal gyrus, inferior parietal gyrus {40} | -53,-30,23 | 18 {40} | Left middle frontal gyrus {8,6} | 31,13,56 | 6 {8,6} |
| SC | VD8 | VD10 | 0.34 | 0.17 | 1.63 | 0.0056 | Right parahippocampal gyrus {37,30} | 61,-42,-25 | 1 {37,30} | Right lobule II (cerebellum) | 10,-49,-29 | 14 {Cbllm} |
| Default network only present in SC | | | | | | | | | | | | |
| SC | VD5 | VD9 | 0.56 | 0.23 | 1.71 | 0.0025 | Right retrosplenial & posterior cingulate cortex {30,23} | 7,-44,14 | 1 {30} | Right angular gyrus, middle occipital gyrus {39,19} | 60,-61,8 | 1 {39} 11-13 {19} |
| SC | VD5 | VD3 | 0.51 | 0.25 | 1.61 | 0.0066 | Right retrosplenial & posterior cingulate cortex {30,23} | 7,-44,14 | 1 {30} | Left parahippocampal gyrus {37,20} | -53,-39,-26 | 1 {37,20} |
| SC | VD1 | VD9 | 0.53 | 0.26 | 1.66 | 0.006 | Left retrosplenial cortex, posterior cingulate {29,30,23} | -7,-45,16 | 1 {30} | Right angular gyrus, middle occipital gyrus {39,19} | 60,-61,8 | 1 {39} 11-13 {19} |
| SC | VD1 | VD3 | 0.49 | 0.22 | 1.66 | 0.0044 | Left retrosplenial cortex, posterior cingulate {29,30,23} | -7,-45,16 | 1 {30} | Left parahippocampal gyrus {37,20} | -53,-39,-26 | 1 {37,20} |
| SC | VD5 | DD4 | 0.51 | 0.17 | 1.77 | 0.0010 | Right retrosplenial & posterior cingulate cortex {30,23} | 7,-44,14 | 1 {30} | Right angular gyrus {39} | 57,-51,32 | 10 {39} |
| SC | DD4 | PD1 | 0.53 | 0.26 | 1.63 | 0.0057 | Right angular gyrus {39} | 57,-51,32 | 10 {39} | Midcingulate cortex, posterior cingulate cortex {23} | 0,-28,34 |  |
| SC | VD5 | PD2 | 0.52 | 0.25 | 1.63 | 0.0058 | Right retrosplenial & posterior cingulate cortex {30,23} | 7,-44,14 | 1 {30} | Precuneus (posterior) {7,19} | 0,-65,46 | 7 {7} |
| SC | LE4 | PD2 | 0.54 | 0.27 | 1.61 | 0.0069 | Left inferior temporal gyrus, middle temporal gyrus {20,37} | -49,-35,-14 | 10 {37} | Precuneus (posterior) {7,19} | 0,-65,46 | 7 {7} |
