# Supplementary material for: Connectivity differences between Gulf War Illness (GWI) phenotypes during a test of attention
Source: PLoS One. 2019 Dec 31;14(12):e0226481. doi: 10.1371/journal.pone.0226481 (PMC6938369; doi:10.1371/journal.pone.0226481)
Supplement: S8 Table — All significant edges in individual groups, pairs of groups, and the entire group were tabulated with the average Fisher’s z-transformed Pearson’s correlation coefficients, standard deviations, Cohen’s d (d > 1.6), and Student’s t-test (FDR < 0.01). Edges were arranged by connected modules (S3 Fig). The anatomical location from Shirer et al. [31], approximated center of mass in Montreal Neurological Institute (MNI) coordinates [140], and most closely aligned BrainMap Intrinsic Connectivity Network (ICN) [94] were estimated for each node. (DOCX) [file pone.0226481.s008.docx]

Table S8. Nodes and edges in START group. All significant edges in individual groups, pairs of groups, and the entire group were tabulated with the average Fisher’s z-transformed Pearson’s correlation coefficients, standard deviations, Cohen’s d (d > 1.6), and Student’s t-test (FDR < 0.01). Edges were arranged by connected modules (Fig S3). The anatomical location from Shirer et al. [31], approximated center of mass in Montreal Neurological Institute (MNI) coordinates [138], and most closely aligned BrainMap Intrinsic Connectivity Network (ICN) [91] were estimated for each node.

| Group | Node 1 | Node 2 | Avg | SD | D | FDR | Node 1 Anatomy | Node 1 MNI | Brain Map20 ICN {BA} | Node 2 Anatomy | Node 2 MNI | Brain Map20 ICN {BA} |
| --- | --- | --- | --- | --- | --- | --- | --- | --- | --- | --- | --- | --- |
| Task network present in START only | | | | | | | | | | | | |
| START | SA3 | SA5 | 0.45 | 0.20 | 1.68 | 0.0022 | Anterior cingulate cortex {24,32}, medial prefrontal cortex {8}, supplementary motor area {6} | 0,11,41 | 4 {24} 6{8,6} 6{24,32} 7 {8} | Right anterior insula {48,47} | 38,30,-8 | 4 {48,47} |
| START | SA3 | BG1 | 0.48 | 0.24 | 1.63 | 0.0037 | Anterior cingulate cortex {24,32}, medial prefrontal cortex {8}, supplementary motor area {6} | 0,11,41 | 4 {24} 6{8,6} 6{24,32} 7 {8} | Left caudate and thalamus | -14,9,4 | 3 |
| START | SA3 | BG2 | 0.47 | 0.24 | 1.62 | 0.004 | Anterior cingulate cortex {24,32}, medial prefrontal cortex {8}, supplementary motor area {6} | 0,11,41 | 4 {24} 6{8,6} 6{24,32} 7 {8} | Right caudate, putamen and thalamus | 14,9,4 | 3 |
| START | SA3 | RE2 | 0.44 | 0.16 | 1.76 | 0.00068 | Anterior cingulate cortex {24,32}, medial prefrontal cortex {8}, supplementary motor area {6} | 0,11,41 | 4 {24} 6{8,6} 6{24,32} 7 {8} | Right middle frontal gyrus {10,46} | 48,49,7 | 7 {46} |
| START | BG2 | VD7 | 0.333 | 0.17 | 1.61 | 0.0043 | Right caudate, putamen and thalamus | 14,9,4 | 3 | Right superior frontal gyrus, middle frontal gyrus {9,8} | 24,39,37 | 6 {9,8} |
| START | RE2 | VD7 | 0.62 | 0.24 | 1.75 | 0.00086 | Right middle frontal gyrus {10,46} | 48,49,7 | 7 {46} | Right superior frontal gyrus, middle frontal gyrus {9,8} | 24,39,37 | 6 {9,8} |
| START | RE2 | RE4 | 0.64 | 0.25 | 1.74 | 0.00099 | Right middle frontal gyrus {10,46} | 48,49,7 | 7 {46} | Right superior frontal gyrus {8} | 12,36,53 | 7 {8} |
| START | SA3 | DAN2 | 0.35 | 0.15 | 1.69 | 0.0018 | Anterior cingulate cortex {24,32}, medial prefrontal cortex {8}, supplementary motor area {6} | 0,11,41 | 4 {24} 6{8,6} 6{24,32} 7 {8} | Left inferior parietal sulcus {2,40,7} | -31,-56,38 | 8 {2} 7 {7} 15 {40} |
| START | SA3 | DAN4 | 0.39 | 0.19 | 1.65 | 0.003 | Anterior cingulate cortex {24,32}, medial prefrontal cortex {8}, supplementary motor area {6} | 0,11,41 | 4 {24} 6{8,6} 6{24,32} 7 {8} | Right inferior parietal lobule {2,40,7} | 27,-60,59 | 8 {2} 7 {7} 15 {40} |
| START | DAN2 | DAN3 | 0.42 | 0.20 | 1.67 | 0.0024 | Left inferior parietal sulcus {2,40,7} | -31,-56,38 | 8 {2} 7 {7} 15 {40} | Right middle frontal gyrus (FEF) {6} | 29,6,60 | 6 {6} |
| START | DAN3 | DAN4 | 0.54 | 0.24 | 1.69 | 0.0019 | Right middle frontal gyrus (FEF) {6} | 29,6,60 | 6 {6} | Right inferior parietal lobule {2,40,7} | 27,-60,59 | 8 {2} 7 {7} 15 {40} |
| START | DAN3 | PD3 | 0.58 | 0.29 | 1.64 | 0.0032 | Right middle frontal gyrus (FEF) {6} | 29,6,60 | 6 {6} | Left angular gyrus {7,40} supramarginal gyrus, superior parietal cortex | -39,-48,47 | 7 {7} |
| Ventrolateral prefrontal cortex network | | | | | | | | | | | | |
| START | SA1 | VD2 | 0.38 | 0.15 | 1.73 | 0.0011 | Left middle frontal gyrus {9,46} | -47,31,23 | 7 {9,46} | Left middle frontal gyrus {8,6} | 31,13,56 | 6 {8,6} |
| START | SA1 | SA4 | 0.55 | 0.24 | 1.70 | 0.0017 | Left middle frontal gyrus {9,46} | -47,31,23 | 7 {9,46} | Right middle frontal gyrus {46,9} | 43,33,17 | 7 {46,9} |
| Default mode network | | | | | | | | | | | | |
| START | DD1 | LE4 | 0.68 | 0.30 | 1.70 | 0.0017 | Medial prefrontal cortex, anterior cingulate cortex, orbitofrontal cortex; Right superior frontal gyrus {9,10,24,32,11} | 0,45,-5 | 6 {9} 4 {24} 2 {10,11} | Left inferior temporal gyrus, middle temporal gyrus {20,37} | -49,-35,-14 | 10 {37} |
| START | LE4 | PD4 | 0.50 | 0.25 | 1.64 | 0.0033 | Left inferior temporal gyrus, middle temporal gyrus {20,37} | -49,-35,-14 | 10 {37} | Right angular gyrus {7,40} supramarginal gyrus, superior parietal cortex | 38,-47,47 | 7 {7} 15 {40} |
| START | RE1 | PD4 | 0.67 | 0.34 | 1.62 | 0.0039 | Right middle frontal gyrus, superior frontal gyrus {46,8,9} | 40,28,43 | 7 {46,8,9} | Right angular gyrus {7,40} supramarginal gyrus, superior parietal cortex | 38,-47,47 | 7 {7} 15 {40} |
