# Supplementary material for: Connectivity differences between Gulf War Illness (GWI) phenotypes during a test of attention
Source: PLoS One. 2019 Dec 31;14(12):e0226481. doi: 10.1371/journal.pone.0226481 (PMC6938369; doi:10.1371/journal.pone.0226481)
Supplement: S9 Table — All significant edges in individual groups, pairs of groups, and the entire group were tabulated with the average Fisher’s z-transformed Pearson’s correlation coefficients, standard deviations, Cohen’s d (d > 1.6), and Student’s t-test (FDR < 0.01). Edges were arranged by connected modules (S3 Fig). The anatomical location from Shirer et al. [31], approximated center of mass in Montreal Neurological Institute (MNI) coordinates [140], and most closely aligned BrainMap Intrinsic Connectivity Network (ICN) [94] were estimated for each node. (DOCX) [file pone.0226481.s009.docx]

Table S9. Nodes and edges in STOPP group. All significant edges in individual groups, pairs of groups, and the entire group were tabulated with the average Fisher’s z-transformed Pearson’s correlation coefficients, standard deviations, Cohen’s d (d > 1.6), and Student’s t-test (FDR < 0.01). Edges were arranged by connected modules (Fig S3). The anatomical location from Shirer et al. [31], approximated center of mass in Montreal Neurological Institute (MNI) coordinates [138], and most closely aligned BrainMap Intrinsic Connectivity Network (ICN) [91] were estimated for each node.

| Group | Node 1 | Node 2 | Avg | SD | d | FDR | | Node 1 Anatomy {BA} | | Node 1 MNI | | Brain Map20 ICN {BA} | | Node 2 Anatomy {BA} | | Node 2 MNI | Brain Map20 ICN {BA} |
| --- | --- | --- | --- | --- | --- | --- | --- | --- | --- | --- | --- | --- | --- | --- | --- | --- | --- |
| Left orbitofrontal gyrus and basal ganglia task network present only in STOPP | | | | | | | | | | | | | | | | | |
| STOPP | LE2 | RE2 | 0.69 | 0.26 | 1.76 | 5.3E-7 | | Left inferior frontal gyrus {10,45}, orbitofrontal gyrus {47} | | -45,42,-3 | | 18 {45, Pars} | | Right middle frontal gyrus {10,46} | | 48,49,7 | 7 {46} |
| STOPP | LE2 | BG2 | 0.46 | 0.20 | 1.69 | 2.5E-6 | | Left inferior frontal gyrus {10,45}, orbitofrontal gyrus {47} | | -45,42,-3 | | 18 {45, Pars} | | Right caudate, putamen and thalamus | | 14,9,4 | 3 |
| STOPP | LE2 | BG1 | 0.42 | 0.21 | 1.63 | 1.1E-5 | | Left inferior frontal gyrus {10,45}, orbitofrontal gyrus {47} | | -45,42,-3 | | 18 {45, Pars} | | Left caudate and thalamus | | -14,9,4 | 3 |
| STOPP | SA5 | BG1 | 0.44 | 0.28 | 1.64 | 9.1E-6 | | Right anterior insula {48,47} | | 38,30,-8 | | 4 {48,47} | | Left caudate and thalamus | | -14,9,4 | 3 |
| Ventrolateral task network | | | | | | |  | |  | |  | |  | |  |  |  |
| STOPP | SA4 | RE4 | 0.62 | 0.31 | 1.63 | 1.1E-5 | | Right middle frontal gyrus {46,9} | | 43,33,17 | | 7 {46,9} | | Right superior frontal gyrus {8} | | 12,36,53 | 7 {8} |
| STOPP | LE1 | RE4 | 0.67 | 0.35 | 1.61 | 1.7E-5 | | Left middle frontal gyrus, superior frontal gyrus (SMA, PMC {8}, DLPFC {9}) | | -29,30,49 | | 6 {8,9} | | Right superior frontal gyrus {8} | | 12,36,53 | 7 {8} |
| STOPP | SA1 | LE1 | 0.71 | 0.33 | 1.66 | 6E-06 | | Left middle frontal gyrus {9,46} | | -47,31,23 | | 7 {9,46} | | Left middle frontal gyrus, superior frontal gyrus (SMA, PMC {8}, DLPFC {9}) | | -29,30,49 | 6 {8,9} |
| Default network | | | | | | | | | | | | | | | | | |
| STOPP | PD2 | DD2 | 0.56 | 0.21 | 1.75 | 5.3E-7 | | Precuneus (posterior) {7,19} | | 0,-65,46 | | 7 {7} | | Left angular gyrus {39} | | -54,-57,33 | 10 {39} |
| STOPP | PD2 | DD3 | 0.68 | 0.25 | 1.76 | 5.3E-7 | | Precuneus (posterior) {7,19} | | 0,-65,46 | | 7 {7} | | Posterior cingulate cortex (PCC), Precuneus (inferior) {23,30} | | 0,-45,20 | 1 {30} |
| STOPP | PD2 | DD4 | 0.48 | 0.19 | 1.73 | 8.2E-7 | | Precuneus (posterior) {7,19} | | 0,-65,46 | | 7 {7} | | Right angular gyrus {39} | | 57,-51,32 | 10 {39} |
| STOPP | PD4 | DD4 | 0.57 | 0.23 | 1.73 | 8.5E-7 | | Right angular gyrus {7,40} supramarginal gyrus, superior parietal cortex | | 38,-47,47 | | 7 {7} 15 {40} | | Right angular gyrus {39} | | 57,-51,32 | 10 {39} |
| STOPP | PD2 | VD9 | 0.75 | 0.35 | 1.66 | 6E-06 | | Precuneus (posterior) {7,19} | | 0,-65,46 | | 7 {7} | | Right angular gyrus, middle occipital gyrus {39,19} | | 60,-61,8 | 1 {39} 11-13 {19} |
| STOPP | VD6 | VD9 | 0.70 | 0.30 | 1.70 | 2E-06 | | Precuneus (superior) {5,7} | | 0,-47,75 | | 9 {5} 7 {7} | | Right angular gyrus, middle occipital gyrus {39,19} | | 60,-61,8 | 1 {39} 11-13 {19} |
| STOPP | RE3 | VD9 | 0.69 | 0.26 | 1.75 | 5.7E-7 | | Right inferior parietal gyrus, supramarginal gyrus, angular gyrus {7,40,39} | | 48,-46,46 | | 7 {7} 10 {39} 15 {40} | | Right angular gyrus, middle occipital gyrus {39,19} | | 60,-61,8 | 1 {39} 11-13 {19} |
| STOPP | RE3 | VD6 | 0.59 | 0.31 | 1.60 | 1.8E-5 | | Right inferior parietal gyrus, supramarginal gyrus, angular gyrus {7,40,39} | | 48,-46,46 | | 7 {7} 10 {39} 15 {40} | | Precuneus (superior) {5,7} | | 0,-47,75 | 9 {5} 7 {7} |
| STOPP | DAN3 | RE3 | 0.76 | 0.36 | 1.66 | 6E-06 | | Right middle frontal gyrus (FEF) {6} | | 29,6,60 | | 6 {6} | | Right inferior parietal gyrus, supramarginal gyrus, angular gyrus {7,40,39} | | 48,-46,46 | 7 {7} 10 {39} 15 {40} |
| Individual edges in STOPP | | | | | | | | | | | | | | | | | |
| STOPP | SP1 | SP2 | 0.71 | 0.35 | 1.63 | 1E-05 | | Left supramarginal gyrus, inferior parietal gyrus {40} | | -53,-30,23 | | 18 {40} | | Right supramarginal gyrus, inferior parietal gyrus {2,40} | | 56,-32,26 | 8 {2} 15 {40} |
| STOPP | PD3 | VD4 | 0.66 | 0.33 | 1.63 | 1.1E-5 | | Left angular gyrus {7,40} supramarginal gyrus, superior parietal cortex | | -39,-48,47 | | 7 {7} | | Left middle occipital gyrus {19,39} | | -53,-66,11 | 11-13 {19} 10 {39} |
| STOPP | LE4 | RE5 | 0.39 | 0.20 | 1.62 | 1.3E-5 | | Left inferior temporal gyrus, middle temporal gyrus {20,37} | | -49,-35,-14 | | 10 {37} | | Left Crus I, Crus II, Lobule VI (cerebellum) | | -38,-58,-32 | 14 {Cbllm} |
