# Supplementary material for: Connectivity differences between Gulf War Illness (GWI) phenotypes during a test of attention
Source: PLoS One. 2019 Dec 31;14(12):e0226481. doi: 10.1371/journal.pone.0226481 (PMC6938369; doi:10.1371/journal.pone.0226481)
Supplement: S10 Table — (DOCX) [file pone.0226481.s010.docx]

S10 Table. Connectivity parameters for nodes for sedentary control group (SC).

| Group | Node | Degree | Betweenness Centrality | Degree Centrality | Closeness Centrality | Current Centrality | Center | Leverage Centrality |
| --- | --- | --- | --- | --- | --- | --- | --- | --- |
| SC | DAN3 | 5 | 0.258 | 0.152 | 0.296 | 0.025 | 1 | 0.180 |
| SC | PD2 | 7 | 0.251 | 0.212 | 0.315 | 0.026 | 0 | 0.255 |
| SC | VD5 | 6 | 0.228 | 0.182 | 0.283 | 0.023 | 0 | 0.248 |
| SC | SA3 | 3 | 0.193 | 0.091 | 0.218 | 0.018 | 0 | 0.067 |
| SC | SP2 | 2 | 0.183 | 0.061 | 0.250 | 0.018 | 0 | -0.314 |
| SC | SA4 | 3 | 0.148 | 0.091 | 0.183 | 0.013 | 0 | 0 |
| SC | VD6 | 4 | 0.147 | 0.121 | 0.311 | 0.024 | 1 | -0.096 |
| SC | PD4 | 5 | 0.116 | 0.152 | 0.311 | 0.024 | 1 | -0.029 |
| SC | PD3 | 6 | 0.111 | 0.182 | 0.303 | 0.025 | 0 | 0.117 |
| SC | VD3 | 3 | 0.102 | 0.091 | 0.226 | 0.018 | 0 | -0.092 |
| SC | DD2 | 4 | 0.101 | 0.121 | 0.271 | 0.022 | 0 | 0.100 |
| SC | SP1 | 3 | 0.095 | 0.091 | 0.257 | 0.021 | 0 | -0.131 |
| SC | DAN1 | 4 | 0.094 | 0.121 | 0.290 | 0.024 | 1 | -0.042 |
| SC | VD2 | 3 | 0.078 | 0.091 | 0.224 | 0.018 | 0 | 0.167 |
| SC | DD3 | 4 | 0.065 | 0.121 | 0.243 | 0.022 | 0 | 0.033 |
| SC | VD4 | 4 | 0.061 | 0.121 | 0.290 | 0.024 | 0 | 0.008 |
| SC | VD8 | 2 | 0.053 | 0.061 | 0.183 | 0.012 | 0 | 0.067 |
| SC | RE1 | 3 | 0.026 | 0.091 | 0.155 | 0.011 | 0 | 0.067 |
| SC | VD7 | 3 | 0.026 | 0.091 | 0.155 | 0.011 | 0 | 0.067 |
| SC | VD9 | 3 | 0.020 | 0.091 | 0.255 | 0.021 | 0 | -0.206 |
| SC | VD1 | 4 | 0.018 | 0.121 | 0.234 | 0.021 | 0 | 0.021 |
| SC | DD4 | 2 | 0.011 | 0.061 | 0.220 | 0.017 | 0 | -0.250 |
| SC | LE3 | 5 | 0.009 | 0.152 | 0.283 | 0.024 | 0 | -0.007 |
| SC | PD1 | 2 | 0.003 | 0.061 | 0.195 | 0.017 | 0 | -0.167 |
| SC | BG1 | 1 | 0.000 | 0.030 | 0.030 |  |  | 0 |
| SC | BG2 | 1 | 0.000 | 0.030 | 0.030 |  |  | 0 |
| SC | LE1 | 1 | 0.000 | 0.030 | 0.179 | 0.012 | 0 | -0.500 |
| SC | LE4 | 1 | 0.000 | 0.030 | 0.234 | 0.015 | 0 | -0.750 |
| SC | RE2 | 1 | 0.000 | 0.030 | 0.209 | 0.014 | 0 | -0.600 |
| SC | RE3 | 4 | 0.000 | 0.121 | 0.277 | 0.023 | 0 | -0.174 |
| SC | RE4 | 2 | 0.000 | 0.061 | 0.133 | 0.010 | 0 | -0.200 |
| SC | SA2 | 1 | 0.000 | 0.030 | 0.030 |  |  | 0 |
| SC | SA5 | 1 | 0.000 | 0.030 | 0.030 |  |  | 0 |
| SC | VD10 | 1 | 0.000 | 0.030 | 0.153 | 0.009 | 0 | -0.333 |
