# Supplementary material for: Connectivity differences between Gulf War Illness (GWI) phenotypes during a test of attention
Source: PLoS One. 2019 Dec 31;14(12):e0226481. doi: 10.1371/journal.pone.0226481 (PMC6938369; doi:10.1371/journal.pone.0226481)
Supplement: S11 Table — (DOCX) [file pone.0226481.s011.docx]

Table S11. Connectivity parameters for edges in the sedentary control (SC) group.

| SC | Node 1 | Node 2 | Edge Betweenness Centrality | Normalized Betweenness Centrality |
| --- | --- | --- | --- | --- |
| SC | PD2 | RE3 | 0.214 | 1.000 |
| SC | DAN3 | SP2 | 0.212 | 0.989 |
| SC | SA3 | SA4 | 0.185 | 0.866 |
| SC | SP2 | SA3 | 0.185 | 0.864 |
| SC | DAN3 | VD6 | 0.129 | 0.601 |
| SC | DAN3 | PD4 | 0.125 | 0.585 |
| SC | VD3 | VD8 | 0.124 | 0.578 |
| SC | PD2 | VD5 | 0.102 | 0.476 |
| SC | SP1 | VD2 | 0.102 | 0.475 |
| SC | VD3 | VD5 | 0.100 | 0.466 |
| SC | DAN1 | PD3 | 0.090 | 0.419 |
| SC | DD2 | PD3 | 0.088 | 0.409 |
| SC | DAN1 | VD6 | 0.082 | 0.381 |
| SC | PD4 | PD2 | 0.082 | 0.381 |
| SC | DD3 | PD1 | 0.073 | 0.339 |
| SC | SA4 | VD7 | 0.072 | 0.337 |
| SC | SA4 | RE1 | 0.072 | 0.337 |
| SC | VD4 | VD6 | 0.068 | 0.318 |
| SC | DD4 | VD5 | 0.058 | 0.270 |
| SC | LE1 | VD2 | 0.052 | 0.241 |
| SC | LE4 | PD2 | 0.052 | 0.241 |
| SC | DD2 | VD4 | 0.052 | 0.241 |
| SC | VD10 | VD8 | 0.052 | 0.241 |
| SC | VD4 | VD9 | 0.050 | 0.234 |
| SC | DAN3 | SP1 | 0.047 | 0.219 |
| SC | SA3 | VD2 | 0.045 | 0.209 |
| SC | DD3 | VD1 | 0.042 | 0.196 |
| SC | PD3 | RE3 | 0.039 | 0.181 |
| SC | DD3 | VD5 | 0.039 | 0.181 |
| SC | DAN1 | SP1 | 0.032 | 0.151 |
| SC | DD2 | RE2 | 0.031 | 0.143 |
| SC | VD7 | RE4 | 0.026 | 0.121 |
| SC | RE1 | RE4 | 0.026 | 0.121 |
| SC | VD1 | VD9 | 0.026 | 0.120 |
| SC | DAN3 | DAN1 | 0.024 | 0.114 |
| SC | PD4 | PD3 | 0.023 | 0.108 |
| SC | DD3 | DD2 | 0.021 | 0.096 |
| SC | VD1 | VD5 | 0.020 | 0.096 |
| SC | VD5 | VD9 | 0.020 | 0.096 |
| SC | LE3 | PD3 | 0.020 | 0.092 |
| SC | PD2 | VD6 | 0.019 | 0.089 |
| SC | VD1 | VD3 | 0.018 | 0.086 |
| SC | LE3 | PD2 | 0.018 | 0.082 |
| SC | PD4 | RE3 | 0.017 | 0.079 |
| SC | LE3 | VD4 | 0.017 | 0.078 |
| SC | PD1 | DD4 | 0.015 | 0.070 |
| SC | PD3 | PD2 | 0.011 | 0.053 |
| SC | LE3 | RE3 | 0.011 | 0.049 |
| SC | LE3 | PD4 | 0.004 | 0.021 |
| SC | SA2 | SA5 | 0.002 | 0.008 |
| SC | VD7 | RE1 | 0.002 | 0.008 |
| SC | BG1 | BG2 | 0.002 | 0.008 |
