# Supplementary material for: Connectivity differences between Gulf War Illness (GWI) phenotypes during a test of attention
Source: PLoS One. 2019 Dec 31;14(12):e0226481. doi: 10.1371/journal.pone.0226481 (PMC6938369; doi:10.1371/journal.pone.0226481)
Supplement: S12 Table — (DOCX) [file pone.0226481.s012.docx]

Table S12. Connectivity parameters for nodes in START phenotype.

| Group | Node | Degree | Betweenness Centrality | Degree Centrality | Closeness Centrality | Current Centrality | Center | Leverage Centrality |
| --- | --- | --- | --- | --- | --- | --- | --- | --- |
| START | PD4 | 6 | 0.286 | 0.182 | 0.398 | 0.034 | 1 | 0.260 |
| START | SA3 | 9 | 0.221 | 0.273 | 0.359 | 0.033 | 0 | 0.446 |
| START | RE1 | 5 | 0.144 | 0.152 | 0.380 | 0.032 | 1 | -0.053 |
| START | VD4 | 6 | 0.140 | 0.182 | 0.340 | 0.033 | 0 | 0.180 |
| START | DD2 | 5 | 0.129 | 0.152 | 0.335 | 0.029 | 0 | 0.104 |
| START | VD9 | 3 | 0.121 | 0.091 | 0.335 | 0.027 | 0 | -0.156 |
| START | VD6 | 7 | 0.120 | 0.212 | 0.354 | 0.033 | 0 | 0.253 |
| START | DAN3 | 6 | 0.117 | 0.182 | 0.335 | 0.033 | 0 | 0.233 |
| START | RE2 | 5 | 0.108 | 0.152 | 0.344 | 0.031 | 0 | -0.035 |
| START | DD4 | 2 | 0.053 | 0.061 | 0.250 | 0.016 | 0 | 0.067 |
| START | LE4 | 2 | 0.053 | 0.061 | 0.283 | 0.018 | 0 | -0.083 |
| START | SA5 | 2 | 0.053 | 0.061 | 0.263 | 0.018 | 0 | -0.152 |
| START | DAN2 | 3 | 0.027 | 0.091 | 0.323 | 0.028 | 0 | -0.278 |
| START | DAN4 | 3 | 0.027 | 0.091 | 0.323 |  |  | -0.278 |
| START | DD3 | 4 | 0.027 | 0.121 | 0.293 | 0.026 | 0 | -0.006 |
| START | PD3 | 4 | 0.019 | 0.121 | 0.293 | 0.029 | 0 | -0.118 |
| START | VD7 | 5 | 0.017 | 0.152 | 0.319 | 0.030 | 0 | 0.015 |
| START | LE3 | 4 | 0.010 | 0.121 | 0.296 | 0.029 | 0 | -0.082 |
| START | DAN1 | 4 | 0.005 | 0.121 | 0.293 | 0.029 | 0 | -0.082 |
| START | SA1 | 2 | 0.004 | 0.061 | 0.068 |  |  | -0.167 |
| START | SP1 | 3 | 0.004 | 0.091 | 0.290 | 0.027 | 0 | -0.292 |
| START | VD2 | 2 | 0.004 | 0.061 | 0.068 |  |  | 0.167 |
| START | BG2 | 3 | 0.001 | 0.091 | 0.263 | 0.025 | 0 | -0.183 |
| START | BG1 | 2 | 0.000 | 0.061 | 0.260 | 0.022 | 0 | -0.418 |
| START | DD1 | 1 | 0.000 | 0.030 | 0.216 | 0.012 | 0 | -0.333 |
| START | LE1 | 1 | 0.000 | 0.030 | 0.045 |  |  | -0.333 |
| START | LE2 | 1 | 0.000 | 0.030 | 0.196 | 0.011 | 0 | -0.333 |
| START | PD2 | 3 | 0.000 | 0.091 | 0.283 | 0.027 | 0 | -0.292 |
| START | RE3 | 1 | 0.000 | 0.030 | 0.277 | 0.017 | 0 | -0.714 |
| START | RE4 | 4 | 0.000 | 0.121 | 0.315 | 0.029 | 0 | -0.179 |
| START | SA2 | 1 | 0.000 | 0.030 | 0.204 | 0.012 | 0 | -0.333 |
| START | SA4 | 1 | 0.000 | 0.030 | 0.045 |  |  | -0.333 |
| START | VD1 | 3 | 0.000 | 0.091 | 0.250 | 0.023 | 0 | -0.131 |
| START | VD5 | 3 | 0.000 | 0.091 | 0.250 | 0.023 | 0 | -0.131 |
