# Supplementary material for: Connectivity differences between Gulf War Illness (GWI) phenotypes during a test of attention
Source: PLoS One. 2019 Dec 31;14(12):e0226481. doi: 10.1371/journal.pone.0226481 (PMC6938369; doi:10.1371/journal.pone.0226481)
Supplement: S13 Table — (DOCX) [file pone.0226481.s013.docx]

Table S13. Connectivity parameters for edges in START phenotype.

| START | Node 1 | Node 2 | Edge Betweenness Centrality | Normalized Betweenness Centrality |
| --- | --- | --- | --- | --- |
| START | PD4 | RE1 | 0.162 | 1.000 |
| START | DD2 | RE2 | 0.119 | 0.736 |
| START | PD4 | VD9 | 0.113 | 0.698 |
| START | LE4 | PD4 | 0.100 | 0.617 |
| START | DD4 | VD9 | 0.100 | 0.617 |
| START | SA3 | SA5 | 0.100 | 0.617 |
| START | PD4 | VD6 | 0.095 | 0.588 |
| START | DD2 | VD4 | 0.083 | 0.512 |
| START | SA3 | RE1 | 0.082 | 0.506 |
| START | SA3 | RE2 | 0.077 | 0.473 |
| START | DAN3 | PD4 | 0.069 | 0.428 |
| START | VD9 | VD4 | 0.067 | 0.411 |
| START | VD4 | VD6 | 0.064 | 0.393 |
| START | DAN4 | DAN3 | 0.052 | 0.323 |
| START | DAN3 | DAN2 | 0.052 | 0.323 |
| START | LE2 | DD4 | 0.052 | 0.319 |
| START | LE4 | DD1 | 0.052 | 0.319 |
| START | PD4 | RE3 | 0.052 | 0.319 |
| START | SA2 | SA5 | 0.052 | 0.319 |
| START | DD3 | VD4 | 0.052 | 0.319 |
| START | DAN4 | SA3 | 0.049 | 0.304 |
| START | DAN2 | SA3 | 0.049 | 0.304 |
| START | SA3 | BG1 | 0.049 | 0.303 |
| START | DAN3 | PD3 | 0.041 | 0.252 |
| START | DD2 | VD1 | 0.036 | 0.220 |
| START | DD2 | VD5 | 0.036 | 0.220 |
| START | SA3 | BG2 | 0.032 | 0.200 |
| START | VD7 | RE1 | 0.030 | 0.184 |
| START | DAN3 | SP1 | 0.029 | 0.179 |
| START | SP1 | VD6 | 0.029 | 0.177 |
| START | RE2 | RE1 | 0.028 | 0.176 |
| START | DAN3 | DAN1 | 0.028 | 0.176 |
| START | DAN1 | VD6 | 0.027 | 0.168 |
| START | PD2 | VD6 | 0.026 | 0.159 |
| START | PD2 | VD4 | 0.022 | 0.138 |
| START | DD3 | DD2 | 0.021 | 0.132 |
| START | RE1 | RE4 | 0.021 | 0.130 |
| START | PD3 | VD6 | 0.021 | 0.127 |
| START | VD7 | RE2 | 0.019 | 0.115 |
| START | VD7 | BG2 | 0.018 | 0.114 |
| START | SA3 | RE4 | 0.015 | 0.095 |
| START | DD3 | VD1 | 0.014 | 0.088 |
| START | DD3 | VD5 | 0.014 | 0.088 |
| START | SA3 | VD7 | 0.014 | 0.084 |
| START | RE2 | RE4 | 0.012 | 0.077 |
| START | SA1 | VD2 | 0.007 | 0.044 |
| START | LE1 | VD2 | 0.005 | 0.033 |
| START | SA1 | SA4 | 0.005 | 0.033 |
| START | DAN1 | PD3 | 0.003 | 0.020 |
| START | BG1 | BG2 | 0.003 | 0.017 |
| START | VD7 | RE4 | 0.003 | 0.017 |
| START | DAN1 | SP1 | 0.002 | 0.015 |
| START | DAN4 | DAN2 | 0.002 | 0.011 |
| START | VD1 | VD5 | 0.002 | 0.011 |
