# Supplementary material for: Connectivity differences between Gulf War Illness (GWI) phenotypes during a test of attention
Source: PLoS One. 2019 Dec 31;14(12):e0226481. doi: 10.1371/journal.pone.0226481 (PMC6938369; doi:10.1371/journal.pone.0226481)
Supplement: S14 Table — (DOCX) [file pone.0226481.s014.docx]

Table S14. Connectivity parameters for nodes in STOPP phenotype.

| Group | Node | Degree | Betweenness Centrality | Degree Centrality | Closeness Centrality | Current Centrality | Center | Leverage Centrality |
| --- | --- | --- | --- | --- | --- | --- | --- | --- |
| STOPP | RE2 | 3 | 0.313 | 0.081 | 0.317 | 0.023 | 1 | -0.264 |
| STOPP | DD2 | 7 | 0.255 | 0.189 | 0.329 | 0.025 | 1 | 0.168 |
| STOPP | RE1 | 5 | 0.252 | 0.135 | 0.265 | 0.017 | 0 | 0.094 |
| STOPP | LE2 | 4 | 0.191 | 0.108 | 0.299 | 0.021 | 1 | 0.155 |
| STOPP | PD2 | 10 | 0.128 | 0.270 | 0.325 | 0.025 | 0 | 0.221 |
| STOPP | DD4 | 4 | 0.107 | 0.108 | 0.302 | 0.023 | 0 | -0.218 |
| STOPP | VD6 | 9 | 0.089 | 0.243 | 0.271 | 0.024 | 0 | 0.192 |
| STOPP | BG1 | 3 | 0.087 | 0.081 | 0.232 | 0.016 | 0 | 0.082 |
| STOPP | RE4 | 5 | 0.083 | 0.135 | 0.216 | 0.015 | 0 | 0.094 |
| STOPP | PD4 | 8 | 0.067 | 0.216 | 0.282 | 0.024 | 0 | 0.113 |
| STOPP | DAN3 | 5 | 0.067 | 0.135 | 0.228 | 0.021 | 0 | 0.035 |
| STOPP | DD3 | 6 | 0.060 | 0.162 | 0.279 | 0.023 | 0 | 0.176 |
| STOPP | PD3 | 7 | 0.051 | 0.189 | 0.292 | 0.024 | 0 | -0.031 |
| STOPP | LE1 | 3 | 0.046 | 0.081 | 0.178 | 0.012 | 0 | 0.150 |
| STOPP | SA5 | 2 | 0.045 | 0.054 | 0.186 | 0.011 | 0 | 0.067 |
| STOPP | SA3 | 5 | 0.041 | 0.135 | 0.215 | 0.015 | 0 | 0.108 |
| STOPP | VD4 | 6 | 0.036 | 0.162 | 0.292 | 0.024 | 0 | -0.101 |
| STOPP | SP1 | 3 | 0.019 | 0.081 | 0.213 | 0.018 | 0 | -0.033 |
| STOPP | RE3 | 7 | 0.019 | 0.189 | 0.265 | 0.024 | 0 | 0.006 |
| STOPP | VD9 | 5 | 0.009 | 0.135 | 0.268 | 0.023 | 0 | -0.181 |
| STOPP | VD2 | 2 | 0.002 | 0.054 | 0.177 | 0.012 | 0 | -0.314 |
| STOPP | LE3 | 6 | 0.001 | 0.162 | 0.257 | 0.023 | 0 | -0.124 |
| STOPP | DAN1 | 2 | 0.001 | 0.054 | 0.183 | 0.016 | 0 | -0.314 |
| STOPP | SP2 | 2 | 0.001 | 0.054 | 0.183 | 0.016 | 0 | -0.314 |
| STOPP | BG2 | 2 | 0.000 | 0.054 | 0.228 | 0.015 | 0 | -0.267 |
| STOPP | DAN2 | 1 | 0.000 | 0.027 | 0.027 |  |  | 0 |
| STOPP | DAN4 | 1 | 0.000 | 0.027 | 0.027 |  |  | 0 |
| STOPP | LE4 | 1 | 0.000 | 0.027 | 0.027 |  |  | 0 |
| STOPP | PD1 | 1 | 0.000 | 0.027 | 0.211 | 0.013 | 0 | -0.714 |
| STOPP | RE5 | 1 | 0.000 | 0.027 | 0.027 |  |  | 0 |
| STOPP | SA1 | 1 | 0.000 | 0.027 | 0.148 | 0.009 | 0 | -0.500 |
| STOPP | SA2 | 1 | 0.000 | 0.027 | 0.153 | 0.008 | 0 | -0.333 |
| STOPP | SA4 | 4 | 0.000 | 0.108 | 0.213 | 0.015 | 0 | -0.056 |
| STOPP | VD1 | 3 | 0.000 | 0.081 | 0.245 | 0.020 | 0 | -0.244 |
| STOPP | VD3 | 1 | 0.000 | 0.027 | 0.027 |  |  | 0 |
| STOPP | VD5 | 3 | 0.000 | 0.081 | 0.245 | 0.020 | 0 | -0.244 |
| STOPP | VD7 | 4 | 0.000 | 0.108 | 0.213 | 0.015 | 0 | -0.083 |
| STOPP | VD8 | 1 | 0.000 | 0.027 | 0.027 |  |  | 0 |
