# Supplementary material for: Connectivity differences between Gulf War Illness (GWI) phenotypes during a test of attention
Source: PLoS One. 2019 Dec 31;14(12):e0226481. doi: 10.1371/journal.pone.0226481 (PMC6938369; doi:10.1371/journal.pone.0226481)
Supplement: S15 Table — (DOCX) [file pone.0226481.s015.docx]

Table S15. Connectivity parameters for edges in STOPP phenotype.

| STOPP | Node 1 | Node 2 | Edge Betweenness Centrality | Normalized Betweenness Centrality |
| --- | --- | --- | --- | --- |
| STOPP | RE2 | RE1 | 0.273 | 1.000 |
| STOPP | RE2 | DD2 | 0.237 | 0.869 |
| STOPP | LE2 | BG2 | 0.126 | 0.461 |
| STOPP | LE2 | BG1 | 0.121 | 0.443 |
| STOPP | LE2 | DD4 | 0.119 | 0.438 |
| STOPP | RE1 | RE4 | 0.107 | 0.391 |
| STOPP | SA5 | BG1 | 0.085 | 0.313 |
| STOPP | LE1 | VD2 | 0.082 | 0.299 |
| STOPP | DD2 | PD2 | 0.076 | 0.277 |
| STOPP | SA3 | SA4 | 0.071 | 0.260 |
| STOPP | DD2 | PD3 | 0.068 | 0.250 |
| STOPP | PD4 | DD4 | 0.058 | 0.213 |
| STOPP | SP1 | VD6 | 0.058 | 0.211 |
| STOPP | DAN3 | PD4 | 0.051 | 0.186 |
| STOPP | DD3 | PD1 | 0.044 | 0.161 |
| STOPP | SA2 | SA5 | 0.044 | 0.161 |
| STOPP | LE1 | RE4 | 0.044 | 0.161 |
| STOPP | PD2 | DD4 | 0.044 | 0.160 |
| STOPP | DD2 | VD4 | 0.043 | 0.158 |
| STOPP | DD3 | PD2 | 0.043 | 0.156 |
| STOPP | SA3 | VD2 | 0.042 | 0.154 |
| STOPP | LE2 | RE2 | 0.040 | 0.146 |
| STOPP | PD2 | VD6 | 0.037 | 0.134 |
| STOPP | DD3 | DD2 | 0.037 | 0.134 |
| STOPP | SA4 | RE4 | 0.036 | 0.130 |
| STOPP | VD7 | RE1 | 0.036 | 0.130 |
| STOPP | VD4 | VD6 | 0.035 | 0.127 |
| STOPP | DAN3 | SP2 | 0.034 | 0.124 |
| STOPP | DAN3 | DAN1 | 0.034 | 0.124 |
| STOPP | DD2 | VD1 | 0.033 | 0.120 |
| STOPP | DD2 | VD5 | 0.033 | 0.120 |
| STOPP | DAN3 | VD6 | 0.031 | 0.112 |
| STOPP | PD2 | RE3 | 0.025 | 0.091 |
| STOPP | PD3 | VD6 | 0.024 | 0.087 |
| STOPP | DD4 | VD9 | 0.023 | 0.086 |
| STOPP | DAN3 | RE3 | 0.021 | 0.078 |
| STOPP | PD4 | PD2 | 0.020 | 0.073 |
| STOPP | PD4 | PD3 | 0.017 | 0.064 |
| STOPP | PD3 | RE3 | 0.016 | 0.060 |
| STOPP | PD2 | VD9 | 0.016 | 0.060 |
| STOPP | DD3 | VD4 | 0.015 | 0.054 |
| STOPP | LE3 | PD2 | 0.013 | 0.049 |
| STOPP | VD6 | VD9 | 0.012 | 0.043 |
| STOPP | DAN1 | SP1 | 0.011 | 0.041 |
| STOPP | SP1 | SP2 | 0.011 | 0.041 |
| STOPP | DD3 | VD1 | 0.010 | 0.036 |
| STOPP | DD3 | VD5 | 0.010 | 0.036 |
| STOPP | LE3 | VD4 | 0.009 | 0.035 |
| STOPP | PD4 | VD6 | 0.009 | 0.033 |
| STOPP | PD2 | VD4 | 0.008 | 0.029 |
| STOPP | LE3 | PD4 | 0.008 | 0.028 |
| STOPP | LE3 | PD3 | 0.007 | 0.025 |
| STOPP | VD9 | RE3 | 0.006 | 0.023 |
| STOPP | PD3 | PD2 | 0.006 | 0.021 |
| STOPP | LE3 | VD6 | 0.005 | 0.019 |
| STOPP | LE1 | SA1 | 0.005 | 0.018 |
| STOPP | SA4 | VD7 | 0.004 | 0.016 |
| STOPP | VD7 | RE4 | 0.004 | 0.016 |
| STOPP | BG1 | BG2 | 0.004 | 0.016 |
| STOPP | PD4 | RE3 | 0.004 | 0.016 |
| STOPP | SA3 | RE4 | 0.004 | 0.013 |
| STOPP | LE3 | RE3 | 0.003 | 0.012 |
| STOPP | PD4 | VD9 | 0.003 | 0.012 |
| STOPP | VD6 | RE3 | 0.003 | 0.012 |
| STOPP | PD3 | VD4 | 0.003 | 0.011 |
| STOPP | SA3 | RE1 | 0.003 | 0.010 |
| STOPP | SA3 | VD7 | 0.003 | 0.010 |
| STOPP | DAN4 | DAN2 | 0.001 | 0.005 |
| STOPP | LE4 | RE5 | 0.001 | 0.005 |
| STOPP | SA4 | RE1 | 0.001 | 0.005 |
| STOPP | VD1 | VD5 | 0.001 | 0.005 |
| STOPP | VD3 | VD8 | 0.001 | 0.005 |
