# Supplementary material for: Connectivity differences between Gulf War Illness (GWI) phenotypes during a test of attention
Source: PLoS One. 2019 Dec 31;14(12):e0226481. doi: 10.1371/journal.pone.0226481 (PMC6938369; doi:10.1371/journal.pone.0226481)
Supplement: S16 Table — (DOCX) [file pone.0226481.s016.docx]

Table S16. Edges with significantly different Fisher z-transformed Pearson correlation coefficients between groups (mean ± SD).

| Edge | SC (n=8) | START (n=9) | STOPP (n=18) |
| --- | --- | --- | --- |
| RAG—MCC (DD4--PD1) | 0.53±0.26 | 0.25±0.20* | 0.22±0.21* |
| LPar—LAG (LE3-PD3) | 1.77±0.48 | 1.26±0.44* | 1.53±0.32 |
| LMOG—PCC (VD4-DD3) | 0.82±0.45 | 0.42±0.22* | 0.53±0.24 |
| LRSC—LPara (VD1-VD3) | 0.49±0.22 | 0.21±0.22* | 0.31±0.21 |
| dACC—RIPS (SA3-DAN4) | 0.01±0.32 | 0.39±0.19* | 0.24±0.33 |
| dACC—LIPS (SA3-DAN2) | -0.07±0.26 | 0.35±0.15* | 0.22±0.36 |
| RBG—RDLPFC (RBG—VD7) | 0.05±0.18 | 0.33±0.17* | 0.18±0.25 |
| RBG—LOFG (RBG—LE2) | 0.26±0.23 | 0.20±0.27† | 0.46±0.2 |
| LPI—LMFG (SP1—VD2) | 0.40±0.20 | 0.13±0.32† | 0.43±0.28 |

*p<0.05 vs. SC or †p<0.05 vs. STOPP by HSD after ANOVA.
