# Supplementary material for: Connectivity differences between Gulf War Illness (GWI) phenotypes during a test of attention
Source: PLoS One. 2019 Dec 31;14(12):e0226481. doi: 10.1371/journal.pone.0226481 (PMC6938369; doi:10.1371/journal.pone.0226481)
Supplement: S1 Fig — Connectivity maps show small communities (“cores”) for (a) RDLPFC task, (b) DAN1 and DAN3, (c) default system, and (d) 3 additional pairs of nodes. These shared edges were indicated by dashed lines on the other figures and were detailed in S6 Table. (e) Ball and spring models of nodes and edges provide a general overview of the complexity of connections during the 0-back task and differences between the 3 groups. The Shirer atlas [31] had irregular regions; therefore estimates of the center of mass for each should be considered very approximate (S1 and S6 Tables). Networks of nodes were visualized using BrainNet Viewer [141]. Montreal Neurological Institute [140] coordinates show x (positive to right), y (positive anterior), z (positive superior), and anatomical left side on the left of each figure. Edges (springs) that were shared by all 3 groups were shown as black lines on the anatomical mesh diagrams. Nodes (balls) were colored for basal ganglia (BG, black), anterior salience (SA, red), posterior salience (SP, magenta), dorsal attention network (DAN, yellow), left executive control network (LE, lime), right executive control network (RE, dark green), dorsal default mode network (DD, cyan), precuneus network (PD, blue), and ventral default mode network (VD, teal). (DOCX) [file pone.0226481.s018.docx]

**Fig S1. Communities of nodes and edges shared by SC, START and STOPP**. Connectivity maps show small communities (“cores”) for (a) RDLPFC task, (b) DAN1 and DAN3, (c) default system, and (d) 3 additional pairs of nodes. These shared edges were indicated by dashed lines on the other figures and were detailed in Table S6. (e) Ball and spring models of nodes and edges provide a general overview of the complexity of connections during the 0-back task and differences between the 3 groups. The Shirer atlas [31] had irregular regions; therefore estimates of the center of mass for each should be considered very approximate (Tables S1 and S6). Networks of nodes were visualized using BrainNet Viewer [139]. Montreal Neurological Institute [138] coordinates show x (positive to right), y (positive anterior), z (positive superior), and anatomical left side on the left of each figure. Edges (springs) that were shared by all 3 groups were shown as black lines on the anatomical mesh diagrams. Nodes (balls) were colored for basal ganglia (BG, black), anterior salience (SA, red), posterior salience (SP, magenta), dorsal attention network (DAN, yellow), left executive control network (LE, lime), right executive control network (RE, dark green), dorsal default mode network (DD, cyan), precuneus network (PD, blue), and ventral default mode network (VD, teal).

| 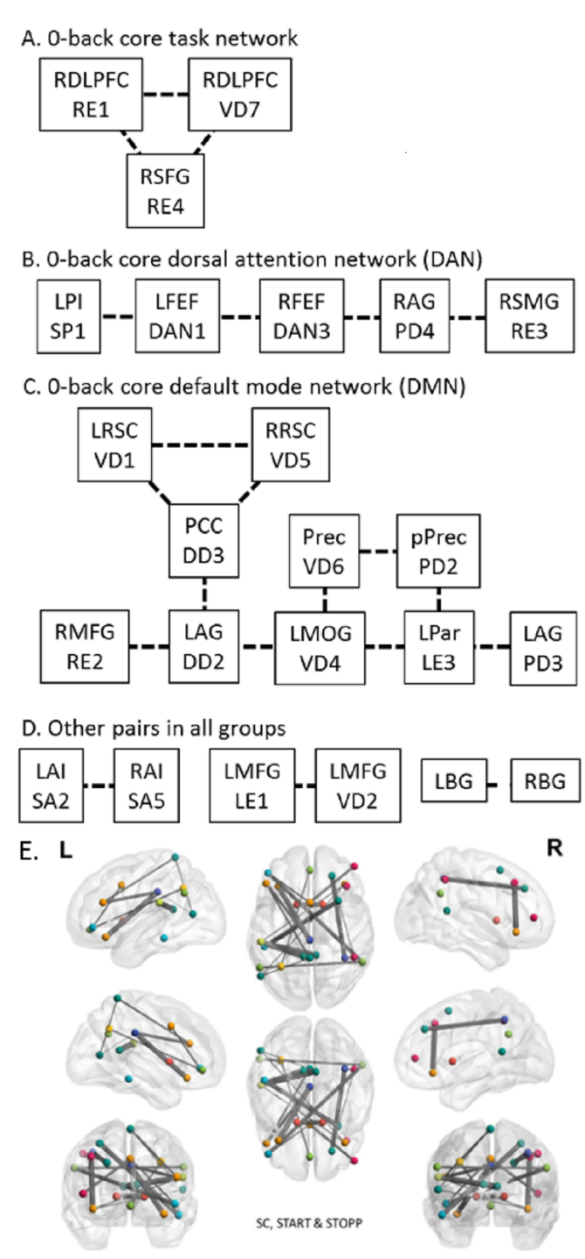 |
| --- |
