# Supplementary material for: Connectivity differences between Gulf War Illness (GWI) phenotypes during a test of attention
Source: PLoS One. 2019 Dec 31;14(12):e0226481. doi: 10.1371/journal.pone.0226481 (PMC6938369; doi:10.1371/journal.pone.0226481)
Supplement: S2 Fig — Edges with d>1.6 and FDR<0.01 that were shared by SC & START (16 edges in 5 communities) (a, b) (S7 Table), SC & STOPP (4 edges in 3 communities) (c, d) (S8 Table), and START & STOPP (16 edges in 5 communities) (e, f) (S9 Table) generally connected frontal to parietal regions, or homoptic regions of the left and right cerebrum. Ball and spring models of nodes and edges are described in the legend for S1 Fig. (DOCX) [file pone.0226481.s019.docx]

Figure S2. Nodes and edges shared by pairs of groups. Edges with d>1.6 and FDR<0.01 that were shared by SC & START (16 edges in 5 communities) (a, b) (Table S7), SC & STOPP (4 edges in 3 communities) (c, d) (Table S8), and START & STOPP (16 edges in 5 communities) (e, f) (Table S9) generally connected frontal to parietal regions, or homoptic regions of the left and right cerebrum. Ball and string models of nodes and edges are described in the legend for Figure S1.

| 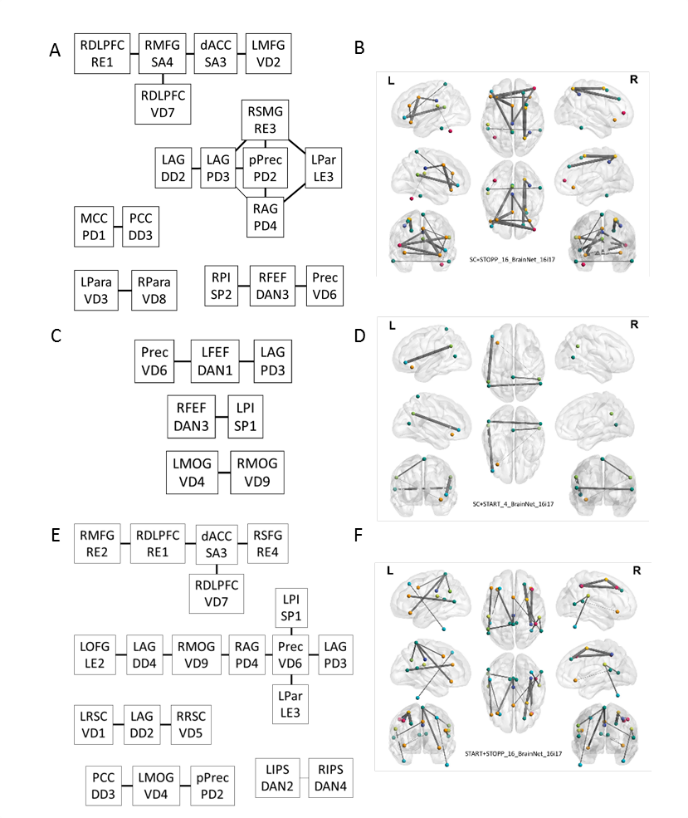 |
| --- |
