# Supplementary material for: Connectivity differences between Gulf War Illness (GWI) phenotypes during a test of attention
Source: PLoS One. 2019 Dec 31;14(12):e0226481. doi: 10.1371/journal.pone.0226481 (PMC6938369; doi:10.1371/journal.pone.0226481)
Supplement: S3 Fig — Edges that were significant exclusively in SC (11 edges, 4 communities) (a, b) (S3 Table), START (16 edges 3 communities) (c, d) (S4 Table), and STOPP (19 edges, 6 communities) (e, f) (S5 Table) were depicted on anatomical and connectivity maps by thick lines (d>1.6 and FDR<0.01). Ball and spring models of nodes and edges are described in the legend for S1 Fig. Colored nodes had high betweenness connectivity or functional importance that was discussed in the text and Table 3. (DOCX) [file pone.0226481.s020.docx]

Figure S3. Unique functional connectivity patterns for each group. Edges that were significant exclusively in SC (11 edges, 4 communities) (a, b) (Table S7), START (16 edges 3 communities) (c, d) (Table S8), and STOPP (19 edges, 6 communities) (e, f) (Table S9) were depicted on anatomical and connectivity maps by thick lines (d>1.6 and FDR<0.01). Ball and spring models of nodes and edges are described in the legend for Figure S1. Colored nodes had high betweenness connectivity or functional importance that was discussed in the text and Table 3.

| 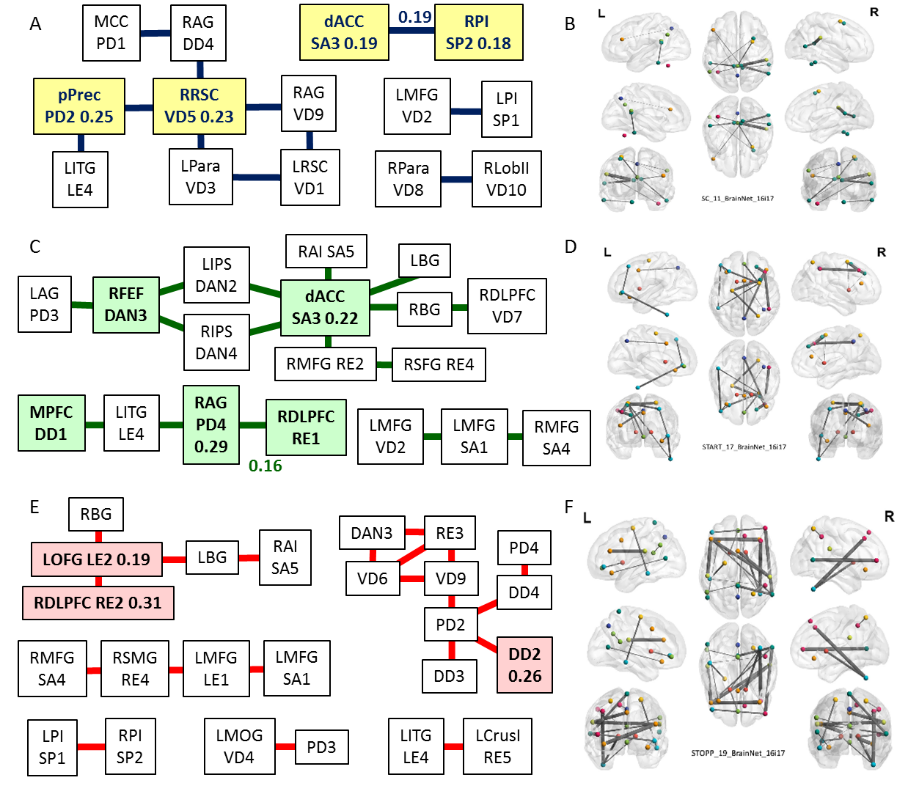 |
| --- |
